# Supplementary material for: Dynamics of polarization-tuned mirror symmetry breaking in a rotationally symmetric system
Source: Nat Commun. 2024 Jul 3;15:5586. doi: 10.1038/s41467-024-49696-x (PMC11222497; doi:10.1038/s41467-024-49696-x)
Supplement: Supplementary file 1 — Supplementary Information [file 41467_2024_49696_MOESM1_ESM.pdf]

Supplementary Information

Dynamics of polarization-tuned mirror symmetry breaking in a rotationally symmetric system

Yu Zhang[1,2†], Zhibin Li[1,2†], Zhen Che[3], Wang Zhang[1,2], Yusen Zhang[1,2], Ziqi Lin[1,2], Zhan Lv[1,2], Chunling Wu[1,2], Longwei Han[1,2], Jieyuan Tang[1,2], Wenguo Zhu[1,2], Yi Xiao[1,2], Huadan Zheng[1,2], Yongchun Zhong[1,2], Zhe Chen[1,2], Jianhui Yu[1,2*]

[1] Key Laboratory of Optoelectronic Information and Sensing Technologies of Guangdong Higher Education Institutes, Department of Optoelectronic Engineering, Jinan University, Guangzhou, 510632, China

[2] Guangdong Provincial Key Laboratory of Optical Fiber Sensing and Communications, Department of Optoelectronic Engineering, Jinan University, Guangzhou, 510632, China

[3] Guangdong Science and Technology Infrastructure Center, Guangzhou 510033, China

† These authors contributed equally: Yu Zhang, Zhibin Li.

* e-mail: jianhuiyu@jnu.edu.cn

Table of Contents

Supplementary Note 1: The LOF for an $SO(2)$ rotationally symmetric object with mirror-symmetric and mirror-asymmetric about the xy-plane.

Supplementary Note 2: The LOF of particle near the interface.

Supplementary Note 3: Dual-dipole model of the LOF on a spherical particle semi-floating at the interface when illuminated by a linearly polarized plane wave.

Supplementary Note 4: Evaluate the integral of the derivative of the Green's function.

Supplementary Note 5: Lateral mirror symmetry breaking of spherical Rayleigh particles.

Supplementary Note 6: LOF of a large-scale particle floating at the air-water interface was calculated by using 3D-Raytracing.

Supplementary Note 7: LOF on Mie lenticular oil droplet floating at the air-water interface.

Supplementary Note 8: Experimental setups.

Supplementary Note 9: LOF efficiency.

Supplementary Note 10: Supplementary video files.

Supplementary References

Supplementary Note 1: The LOF for an *SO*(2) rotationally symmetric object with mirror-symmetric and mirror-asymmetric about the *xy*-plane.

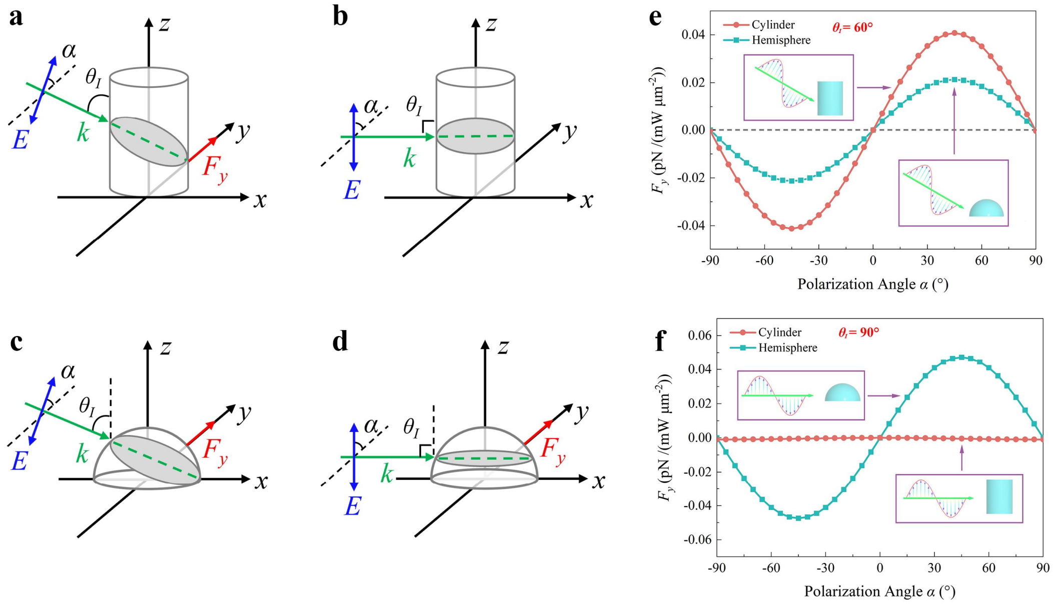

Supplementary Figure 1. (**a**) For an *SO*(2) rotationally symmetric object that is mirror-symmetric about the *xy*-plane, the mirror symmetry of the light-matter interaction about the *xy*-plane is broken by an obliquely incident (0°< θ_I < 90°) linearly polarized plane wave. The lateral optical force (LOF) can be generated by the diagonally polarized light (deviates from *s*-or *p*-polarization). (**b**) For an *SO*(2) rotationally symmetric object that is mirror-symmetric about the *xy*-plane, the mirror symmetry of the light-matter interaction about the *xy*-plane is maintained when the incident angle θ_I = 90°. The LOF disappears regardless of polarization orientation. (**c-d**) For an *SO*(2) rotationally symmetric object with mirror-asymmetric about the *xy*-plane, when the incident light is incident obliquely (0°< θ_I < 90°) or vertically (θ_I = 90°), LOF can be induced when the polarization angle deviates from *s*- or *p*-polarization. (**e-f**) The dependence of the LOF of the cylinder and hemisphere on polarization angle when the incident angle is 60° (**e**) and 90° (**f**), respectively.

In the main text, we proposed a general mirror symmetry breaking (MSB) mechanism that uses a single linearly polarized (LP) plane wave to induce MSB of light scattering and generate lateral optical force (LOF) in an *SO*(2) rotationally symmetric object. The MSB mentioned in our work refers to the MSB of the scattering field with respective to the *xz*-plane, where the particle is mirror-symmetric about the *xz*-plane. Therefore, the LOF caused by the MSB is along the *y*-axis. The proposed mechanism only needs to satisfy the following two conditions: (1) the object has *SO*(2) rotational symmetry, that is, the object is mirror-symmetric about the *xz*-plane, (2) the oblique incidence of a diagonally polarized plane wave. To verify the MSB mechanism, six different objects with the *SO*(2) rotational symmetry in Fig. 1a and Fig. 2a in the main text are demonstrated to break mirror symmetry only by diagonal polarization. Here, these objects are classified into two types: (1) the mirror-symmetric object about the

xy-plane, such as a cylinder; (2) the mirror-asymmetric object about the *xy*-plane, such as a hemisphere, a cone, and a spherical particle semi-floating at the air-water interface.

For the object with mirror symmetry about the *xy*-plane, an oblique incidence of light (0°< θ_I <90°) becomes an essential condition to break the *xy*-plane mirror symmetry and generate the LOF by diagonal polarization, such as a cylinder as shown in Supplementary Fig. 1a and Supplementary Fig. 1e. When the incidence of light becomes vertical (θ_I = 90°), the mirror symmetry cannot be broken by the diagonal polarization and the LOF disappears for the object with the *xy*-plane mirror symmetry, as shown in Supplementary Fig. 1b and Supplementary Fig. 1f. However, for the object with mirror asymmetry about the *xy*-plane (such as a hemisphere), the vertical incidence of light can also break the *xz*-plane mirror symmetry by the diagonal polarization and generate the LOF along the *y* direction, as shown in Supplementary Fig. 1d and Supplementary Fig. 1f. In particular, when the incident light is vertical (θ_I = 90°), the maximum LOF efficiency of the hemisphere with a radius of 500 nm is 2.2 times higher than that for the oblique incidence (θ_I = 60°). This indicates that, for the object with the *xy*-plane mirror asymmetry, the degree of MSB induced by the vertical illumination is higher than that for the oblique illumination.

In summary, for an *SO*(2) rotationally symmetric object with the *xy*-plane mirror symmetry, the oblique incident of light is an essential condition to break the *xz*-plane mirror symmetry of the light-matter system. For the object with the mirror asymmetry to the *xy*-plane, the diagonal polarization of the incident light (even at vertical incidence) could break the *xz*-plane mirror symmetry and generate the LOF along the *y* direction. Therefore, the breaking of *xy*-plane mirror symmetry becomes one of the essential conditions to generate the LOF.

Supplementary Note 2: The LOF of particle near the interface.

Supplementary Fig. 2 shows the LOFs for a polystyrene (PS) particle (n_p = 1.5983 @ λ = 532 nm) completely immersed in the air, in the water and semi-floating at the air-water interface, respectively. The particle radius is 0.5 μm, and when the particle is completely immersed in air or water, the distance from the particle to the interface is 0.5 μm. Firstly, it is seen that in all three cases, the LOF varies sinusoidally with polarization angle α, i.e., LOF is proportional to sin(2α). Secondly, when a particle is semi-floating at the air-water interface, the maximum LOF is 0.073 pN/(mW μm^{-2}), which is 28 times higher than that when the particle is completely in the air and 7.3

times higher than that when the particle is completely in the water. Therefore, the MSB of polarization-induced semi-floating particle light scattering not only provides an ultra-highly efficient mechanism for the LOF but also a highly tunable method for the amplitude and direction of the LOF.

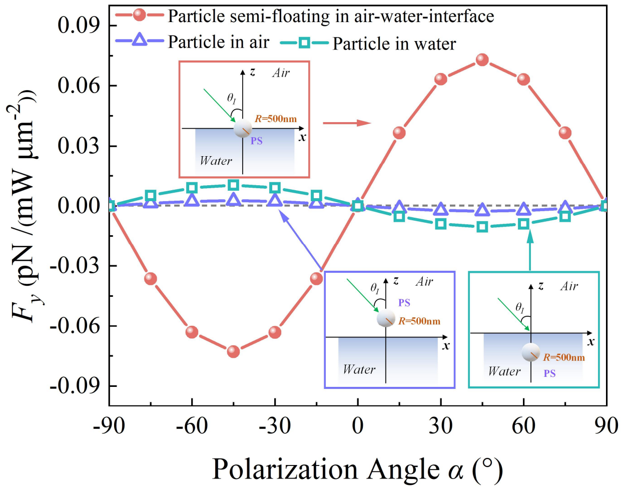

Supplementary Figure 2. The dependence of the lateral optical force on the polarization angle for particles completely in the air, water, and semi-floating at the air-water interface is numerically calculated by the 3D-FDTD method.

Supplementary Note 3: Dual-dipole model of the LOF on a spherical particle semi-floating at the interface when illuminated by a linearly polarized plane wave.

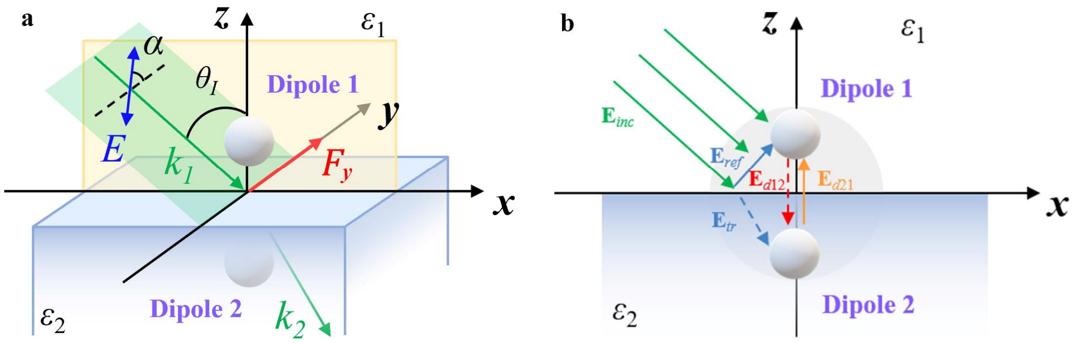

Supplementary Figure 3. Schematic diagram of the dual-dipole model. (**a**) The two dipoles located in semi-infinite transparent medium 1 and medium 2, respectively, are illuminated by an LP plane wave. Due to the presence of the interface, the polarization breaks the mirror symmetry with respect to the *xz*-plane, thus leading to an LOF in the *y* direction on the two dipoles. (**b**) The LOF mainly arises from the interaction between dipole 1 and dipole 2 through their inter-scattering.

To reveal the physical origin of the polarization-induced MSB and the LOF in an *SO*(2) rotational symmetry system, a dual-dipole model is established to derive the LOF F_y acting on the isotropic spherical object semi-floating at the interface between two mediums when illuminating with an LP plane wave, as shown in Supplementary Fig.

3a. Here, the LOF F_y in the y direction perpendicular to the incident plane xz-plane arises from the interaction of the light scattering between the two dipoles, as illustrated in Supplementary Fig. 3b. Dipole 1 at $\mathbf{r}_1 = (x_0, y_0, z_1)$ and dipole 2 at $\mathbf{r}_2 = (x_0, y_0, z_2)$ are respectively located in semi-infinite transparent medium 1 and medium 2. An LP plane wave with an amplitude E_{inc} polarized at angle α is incident from medium 1 to medium 2. We denote the dielectric permittivity of medium 1 and medium 2 as (ε_1, ε_2), respectively.

The total LOF F_y acting on the two dipoles can be expressed in a general way[1]

$$\langle F_y \rangle = \langle F_y^{d1} \rangle + \langle F_y^{d2} \rangle = \frac{1}{2}\mathrm{Re}\left(\mathbf{d}_1 \partial_y \mathbf{E}_1^* + \mathbf{d}_2 \partial_y \mathbf{E}_2^*\right), \quad (1)$$

where F_y^{di} represent the LOF acting on the dipole i (i=1,2). $\mathbf{d}_i = \alpha_{e,i}\mathbf{E}_i$ is the induced electric dipole moment, $\alpha_{e,i}$ is polarizability, $\mathbf{E}_i$ is the local field at the location of the dipole i. The symbol ∂_y denotes the partial derivative with respect to the transversal coordinate y. The electric field $\mathbf{E}_{di}(\mathbf{r})$ produced by dipole $\mathbf{d}_i$ located at $\mathbf{r}_i = (x_0, y_0, z_i)$ can be expressed through dyadic Green's function[2]

$$\mathbf{E}_{di}(\mathbf{r}) = \omega^2 \mu_0 \overleftrightarrow{\mathbf{G}}(\mathbf{r}, \mathbf{r}_i)\mathbf{d}_i(\mathbf{r}_i). \quad (2)$$

Here ω is the frequency of the incident wave, and μ_0 is the vacuum dielectric permeability.

At the dipole 1 location, the local field is $\mathbf{E}_1 = \mathbf{E}_{inc} + \mathbf{E}_{ref} + \mathbf{E}_{d21}$, as shown by the solid line in Supplementary Fig. 3b. Here, $\mathbf{E}_{inc}$ and $\mathbf{E}_{ref}$ are the incident field and the reflected field from the interface. $\mathbf{E}_{d21}$ is the dipole field transmitted from dipole 2 to the location of dipole 1. Since the incident and reflected fields are independent on a lateral coordinate y, the derivative in Eq. (1) is $\partial_y \mathbf{E}_1 = \partial_y \mathbf{E}_{d21}$ and can be expressed with Green's functions as

$$\partial_y \mathbf{E}_{d21} = \omega^2 \mu_0 \partial_y \overleftrightarrow{\mathbf{G}}_2^{tr}(\mathbf{r}_1, \mathbf{r}_2)\mathbf{d}_2(\mathbf{r}_2). \quad (3)$$

In Eq. (3), $\overleftrightarrow{\mathbf{G}}_2^{tr}$ is the transmission Green's function of the dipole 2. The derivatives can be expressed as[2]

$$\partial_y \overleftrightarrow{\mathbf{G}}_2^{tr}(\mathbf{r}_1, \mathbf{r}_2) = -\frac{1}{8\pi^2}\int_{-\infty}^{\infty}\int_{-\infty}^{\infty} k_y \left(\overleftrightarrow{\mathbf{M}}_{2,tr}^{s} + \overleftrightarrow{\mathbf{M}}_{2,tr}^{p}\right)\exp\left[i\left(k_{z1}z_1 - k_{z2}z_2\right)\right]dk_x dk_y \ , \quad (4)$$

$$\overleftrightarrow{\mathbf{M}}_{2,tr}^{s} = \frac{t_2^s(k_x, k_y)}{k_{z2}\left(k_x^2 + k_y^2\right)}\begin{pmatrix} k_y^2 & -k_x k_y & 0 \\ -k_x k_y & k_x^2 & 0 \\ 0 & 0 & 0 \end{pmatrix}, \quad (5)$$

$$\overleftrightarrow{\mathbf{M}}_{2,tr}^{p} = \frac{t_2^p\left(k_x,k_y\right)}{k_1k_2\left(k_x^2+k_y^2\right)}\begin{pmatrix} k_x^2k_{z1} & k_xk_yk_{z1} & k_x\left(k_x^2+k_y^2\right)k_{z1}/k_{z2} \\ k_xk_yk_{z1} & k_y^2k_{z1} & k_y\left(k_x^2+k_y^2\right)k_{z1}/k_{z2} \\ k_x\left(k_x^2+k_y^2\right) & k_y\left(k_x^2+k_y^2\right) & \left(k_x^2+k_y^2\right)^2/k_{z2} \end{pmatrix}. \tag{6}$$

Here, t_2^s, t_2^p are the Fresnel transmission coefficients for *s*- and *p*-polarized wave from medium 2 to medium 1. The longitudinal wavenumber is given by $k_{zi}=\sqrt{k_i^2-(k_x^2+k_y^2)}$, i=1,2. k_1 and k_2 are the wavenumbers in a medium where the dipole is located. For the transmission Green's function $\overleftrightarrow{\mathbf{G}}_2^{tr}$, only $\overleftrightarrow{\mathrm{M}}_{2,tr}^{p,yz}$ and $\overleftrightarrow{\mathrm{M}}_{2,tr}^{p,zy}$ terms remain nonzero after integration. Then, substituting Eqs. (3) and (4) into Eq. (1), the LOF exerted on dipole 1 can be expressed as

$$\left\langle F_y^{d1}\right\rangle = \left\langle F_y^{d21}\right\rangle = \frac{k_0^2}{2\varepsilon_0}\mathrm{Re}\left(d_{1z}^*d_{2y}\partial_y\overleftrightarrow{G}_{2,zy}^{tr} + d_{1y}^*d_{2z}\partial_y\overleftrightarrow{G}_{2,yz}^{tr}\right), \tag{7}$$

where k_0 is the wavenumber in vacuum, and ε_0 is permittivity in vacuum.

Similarly, for the dipole 2, the local field is $\mathbf{E}_2 = \mathbf{E}_{tr} + \mathbf{E}_{d12}$, as shown by the dashed line in Supplementary Fig. 3b. Here, $\mathbf{E}_{tr}$ is the optical field transmitted through the interface from the incident field $\mathbf{E}_{inc}$, and $\mathbf{E}_{d12}$ is the dipole field transmitted from the dipole 1 to the location of the dipole 2. Since the transmitted field $\mathbf{E}_{tr}$ is also independent on the lateral coordinate y, and thus the derivative in Eq. (1) is reduced to $\partial_y\mathbf{E}_2 = \partial_y\mathbf{E}_{\mathrm{d}12}$ and can be expressed as

$$\partial_y\mathbf{E}_{d12} = \omega^2\mu_0\partial_y\overleftrightarrow{\mathbf{G}}_1^{tr}\left(\mathbf{r}_2,\mathbf{r}_1\right)d_1\left(\mathbf{r}_1\right). \tag{8}$$

In Eq. (8), $\overleftrightarrow{\mathbf{G}}_1^{tr}$ is the transmission Green's function of the dipole 1. For the same sake as the discussed above, only *yz* and *zy* elements of the $\overleftrightarrow{\mathbf{G}}_1^{tr}$ remain nonzero. Substituting Eq. (8) into Eq. (1), one can obtain the LOF exerted on the dipole 2

$$\left\langle F_y^{d2}\right\rangle = \left\langle F_y^{d12}\right\rangle = \frac{k_0^2}{2\varepsilon_0}\mathrm{Re}\left(d_{2z}^*d_{1y}\partial_y\overleftrightarrow{G}_{1,zy}^{tr} + d_{2y}^*d_{1z}\partial_y\overleftrightarrow{G}_{1,yz}^{tr}\right). \tag{9}$$

Consequently, the total LOF induced by linear polarization from the interaction between the dipole scattering fields of dipole 1 and dipole 2 can be expressed as

$$\begin{aligned}\left\langle F_y\right\rangle &= \left\langle F_y^{d21}\right\rangle + \left\langle F_y^{d12}\right\rangle \\ &= \frac{k_0^2}{2\varepsilon_0}\mathrm{Re}\left(d_{1z}^*d_{2y}\partial_y\overleftrightarrow{G}_{2,zy}^{tr} + d_{1y}^*d_{2z}\partial_y\overleftrightarrow{G}_{2,yz}^{tr} + d_{2z}^*d_{1y}\partial_y\overleftrightarrow{G}_{1,zy}^{tr} + d_{2y}^*d_{1z}\partial_y\overleftrightarrow{G}_{1,yz}^{tr}\right),\end{aligned} \tag{10}$$

where, $\partial_y\overleftrightarrow{G}_{1,yz}^{tr}$, $\partial_y\overleftrightarrow{G}_{1,zy}^{tr}$, $\partial_y\overleftrightarrow{G}_{2,yz}^{tr}$ and $\partial_y\overleftrightarrow{G}_{2,zy}^{tr}$ can be expressed as

$$\partial_y\overleftrightarrow{G}_{2,zy}^{tr} = -\frac{1}{8\pi k_1k_2}\int_0^\infty t_2^p\left(k_\rho\right)k_\rho^3\exp\left[i\left(\sqrt{k_1^2-k_\rho^2}z_1 - \sqrt{k_2^2-k_\rho^2}z_2\right)\right]dk_\rho, \tag{11}$$

$$\partial_y \overleftrightarrow{G}^{tr}_{2,yz} = -\frac{1}{8\pi k_1 k_2}\int_0^{\infty} \frac{\sqrt{k_1^2 - k_\rho^2}}{\sqrt{k_2^2 - k_\rho^2}} t_2^p\left(k_\rho\right) k_\rho^3 \exp\left[i\left(\sqrt{k_1^2 - k_\rho^2} z_1 - \sqrt{k_2^2 - k_\rho^2} z_2\right)\right] dk_\rho \ , \quad (12)$$

$$\partial_y \overleftrightarrow{G}^{tr}_{1,zy} = -\frac{1}{8\pi k_1 k_2}\int_0^{\infty} t_1^p\left(k_\rho\right) k_\rho^3 \exp\left[i\left(\sqrt{k_1^2 - k_\rho^2} z_1 - \sqrt{k_2^2 - k_\rho^2} z_2\right)\right] dk_\rho \ , \quad (13)$$

$$\partial_y \overleftrightarrow{G}^{tr}_{1,yz} = -\frac{1}{8\pi k_1 k_2}\int_0^{\infty} \frac{\sqrt{k_2^2 - k_\rho^2}}{\sqrt{k_1^2 - k_\rho^2}} t_1^p\left(k_\rho\right) k_\rho^3 \exp\left[i\left(\sqrt{k_1^2 - k_\rho^2} z_1 - \sqrt{k_2^2 - k_\rho^2} z_2\right)\right] dk_\rho \ . \quad (14)$$

Supplementary Note 4: Evaluate the integral of the derivative of the Green's function.

To further understand how the incident angle θ_I and polarization angle α of LP plane wave affect the magnitude and direction of the total LOF, the integral of the derivative of the Green's function should be considered. For convenience, we perform the substitutions $s=k_\rho/k_2$, and the Fresnel transmission coefficient t_1^p and t_2^p can be expressed as[2,3]

$$t_1^p\left(s\right) = \frac{2\sqrt{\varepsilon_{12} - s^2}}{\sqrt{\varepsilon_{12} - s^2} + \varepsilon_{12}\sqrt{1 - s^2}} \sqrt{\varepsilon_{12}} \ , \quad (15)$$

$$t_2^p\left(s\right) = \frac{2\sqrt{1 - s^2}}{\sqrt{\varepsilon_{12} - s^2} + \varepsilon_{12}\sqrt{1 - s^2}} \sqrt{\varepsilon_{12}} \ . \quad (16)$$

Since the effective phase retardation between the two dipoles is mainly contributed to force, we can assume the positions of two dipoles have the relationship z_2=-$\varepsilon_{12} z_1$ (z_1>0) without loss of the generality. Using Eqs. (15) and (16) and $\xi = k_2 z_1$, the integral term of Eqs. (11-14) can be rewritten as

$$\partial_y \overleftrightarrow{G}^{tr}_{2,zy} = \partial_y \overleftrightarrow{G}^{tr}_{1,yz} = -\frac{k_2^2}{4\pi} I_1\left(\varepsilon_{12}, \xi\right) \ , \quad (17)$$

$$\partial_y \overleftrightarrow{G}^{tr}_{2,yz} = \partial_y \overleftrightarrow{G}^{tr}_{1,zy} = -\frac{k_2^2}{4\pi} I_2\left(\varepsilon_{12}, \xi\right) \ . \quad (18)$$

The integral term is replaced by $I_i(\varepsilon_{12},\xi)$ in Eqs. (17-18)

$$I_1\left(\varepsilon_{12}, \xi\right) = \int_0^{\infty} \frac{s^3\sqrt{1 - s^2}}{\sqrt{\varepsilon_{12} - s^2} + \varepsilon_{12}\sqrt{1 - s^2}} \exp\left[i\xi\left(\sqrt{\varepsilon_{12} - s^2} + \varepsilon_{12}\sqrt{1 - s^2}\right)\right] ds \ , \quad (19)$$

$$I_2\left(\varepsilon_{12}, \xi\right) = \int_0^{\infty} \frac{s^3\sqrt{\varepsilon_{12} - s^2}}{\sqrt{\varepsilon_{12} - s^2} + \varepsilon_{12}\sqrt{1 - s^2}} \exp\left[i\xi\left(\sqrt{\varepsilon_{12} - s^2} + \varepsilon_{12}\sqrt{1 - s^2}\right)\right] ds \ . \quad (20)$$

Therefore, the final expression for the total LOF (Eq. (10)) can be rewritten as

$$\left\langle F_y \right\rangle = \frac{k_0^2}{\varepsilon_0}\left[\mathrm{Re}\left(\partial_y \overleftrightarrow{G}^{tr}_{1,yz}\right)\mathrm{Re}\left(d_{2y} d_{1z}^*\right) + \mathrm{Re}\left(\partial_y \overleftrightarrow{G}^{tr}_{2,yz}\right)\mathrm{Re}\left(d_{1y}^* d_{2z}\right)\right] \ . \quad (21)$$

Using the linear polarization of the incident light and Fresnel transmission coefficients for s- and p- polarization, the magnitude of the dipole moments in Eq. (21) are obtained

$$d_{1z}^{*}d_{2y}=\frac{\alpha_{e,1}^{*}\alpha_{e,2}}{2}\left|E_{inc}\right|^{2}t_{s}\left(\theta_{I}\right)\sin\left(2\alpha\right)\sin\left(\theta_{I}\right)\left\{\begin{array}{l}\cos\left(\delta_{d12}\right)+r_{p}^{*}\left(\theta_{I}\right)\cos\left(\delta_{ref}-\delta_{d12}\right)\\+i\left[\sin\left(\delta_{d12}\right)-r_{p}^{*}\left(\theta_{I}\right)\sin\left(\delta_{ref}-\delta_{d12}\right)\right]\end{array}\right\},$$
$$d_{1y}^{*}d_{2z}=\frac{\alpha_{e,1}^{*}\alpha_{e,2}}{2}\sqrt{\varepsilon_{12}}\left|E_{inc}\right|^{2}t_{p}\left(\theta_{I}\right)\sin\left(2\alpha\right)\sin\left(\theta_{I}\right)\left\{\begin{array}{l}\cos\left(\delta_{d12}\right)+r_{s}^{*}\left(\theta_{I}\right)\cos\left(\delta_{ref}-\delta_{d12}\right)\\+i\left[\sin\left(\delta_{d12}\right)-r_{s}^{*}\left(\theta_{I}\right)\sin\left(\delta_{ref}-\delta_{d12}\right)\right]\end{array}\right\}. \quad (22)$$

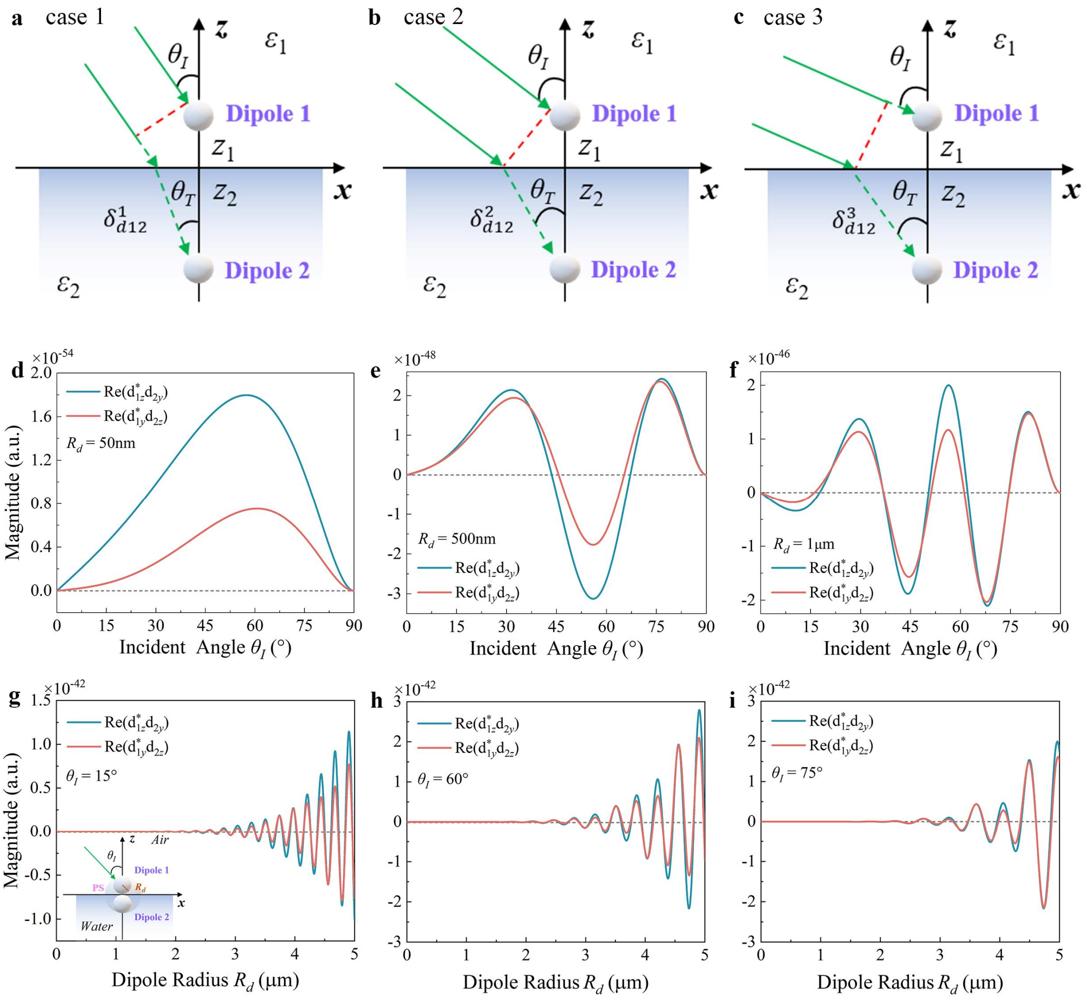

Supplementary Figure 4. (**a-c**) Three cases of phase retardation between the dipole 1 and the dipole 2. (**d-f**) The dependence of the real part of the dipole moment terms on the angle of incidence for particles with radii of 50 nm, 500 nm and 1000 nm, respectively. (**g-i**) The dependence of the real part of the dipole moment terms on the radius of particles at $\theta_I = 15°$, 60° and 75°, respectively. The inset shows the dual-dipole model, where the dipole radius $R_d = |z_1| = |z_2|$ is half of the PS particle radius R.

In Eq. (22), $t_s(\theta_I)$ and $t_p(\theta_I)$ are the amplitude transmission coefficients for s- and p-polarized waves, respectively. $\varepsilon_{12}=\varepsilon_1/\varepsilon_2$ is the relative permittivity of two media. $\delta_{ref}=2k_1z_1\cos(\theta_I)$ is the phase retardation between the incident field $\mathbf{E}_{inc}$ and the reflected field $\mathbf{E}_{ref}$, and δ_{d12} is the phase retardation between the dipole 1 and the dipole

2. Here, δ_{d12} has three cases depending on the distance of the two dipoles from the interface and the incident angle, as shown in Supplementary Figs. 4a-4c, where θ_T is the refracted angle. According to the schematic diagram in Supplementary Figs. 4a-4c, the three kinds of phase retardations δ_{d12} can be expressed as

$$\delta_{d12}^{1}=\delta_{d12}^{3}=k_2\left[|z_1|\sqrt{\varepsilon_{12}}\cos(\theta_I)+|z_2|\sqrt{1-\varepsilon_{12}\sin^2(\theta_I)}\right], \tag{23}$$

$$\delta_{d12}^{2}=\frac{k_2|z_2|}{\sqrt{1-\varepsilon_{12}\sin^2(\theta_I)}}, \quad |z_2|\tan(\theta_I)\tan(\theta_T)=|z_1|. \tag{24}$$

It is seen that case 1 and case 3 have the same phase retardation and it is a function of dipole position and incident angle θ_I. The case 2 is a special case at a special angle of incidence, which satisfying $|z_2|\tan(\theta_I)\tan(\theta_T)=|z_1|$.

Consider the case in the main text, where the incident light is incident from the air (n_1 = 1) into the water (n_2 = 1.337), a PS particle (n_p = 1.5983 @ λ = 532 nm) is semi-floating at the air-water interface, the Fresnel coefficients for s- and p-polarized waves are all real values. If the dipole absorption is ignored ($\mathrm{Im}(\alpha_e)=0$), the real part of the dipole moment terms in Eq. (22) can be simplified as

$$\mathrm{Re}\left(d_{1z}^{*}d_{2y}\right)=\frac{\alpha_{e,1}\alpha_{e,2}|E_{inc}|^2 t_s(\theta_I)\sin(2\alpha)\sin(\theta_I)}{2}\left[\cos(\delta_{d12})+r_p(\theta_I)\cos(\delta_{ref}-\delta_{d12})\right] \tag{25}$$

$$\mathrm{Re}\left(d_{1y}^{*}d_{2z}\right)=\frac{\alpha_{e,1}\alpha_{e,2}\sqrt{\varepsilon_{12}}|E_{inc}|^2 t_p(\theta_I)\sin(2\alpha)\sin(\theta_I)}{2}\left[\cos(\delta_{d12})+r_s(\theta_I)\cos(\delta_{ref}-\delta_{d12})\right] \tag{26}$$

According to Eqs. (23-26), the phase retardations δ_{ref} and δ_{d12} render the magnitude of the dipole momentum terms oscillatory with the incident angle θ_I and dipole radius R_d. Here, the dipole radius R_d is half of the PS particle radius R, which can be approximately considered equivalent to the dipole distances $|z_1|$ and $|z_2|$ away from the air-water interface ($R_d=|z_1|=|z_2|$), as shown in the dual-dipole model shown in the inset in Supplementary Fig. 4g. The oscillatory dependence of the dipole moment terms on incident angle θ_I and dipole radius R_d is calculated and shown in Supplementary Figs. 4d-4f and Supplementary Figs. 4g-4i, respectively. Supplementary Figs. 4d-4f for R_d = 50 nm, 500 nm, and 1 μm show that the oscillatory variation with incident angle θ_I become more rapid for a larger radius R_d, that is the oscillatory periodic becomes shorter for a larger radius. This is due to the cosine function of the phase retardation and its proportion to the dipole distances $|z_1|$ and $|z_2|$ in Eqs. (22), (25) and (26). Similarly, Supplementary Figs. 4g-4i for θ_I = 15°, 60°, 75° show that the

oscillatory variation with the radius R_d turns more rapid for a smaller incident angle because of the phase retardation δ_{d12} proportional to the term $k_1R_d\cos(\theta_I)+k_2R_d\cos(\theta_T)$ ($R_d=|z_1|=|z_2|$) in Eq. (23). Additionally, in Supplementary Figs. 4g-4i, the magnitude of the dipole terms in Eqs. (25-26) will increase with radius R_d. According to the Clausius-Mossotti relationship $\alpha_{e,i}=4\pi\varepsilon_i R_d^3\left[\left(\varepsilon_p\text{-}\varepsilon_i\right)/\left(\varepsilon_p+2\varepsilon_i\right)\right]$ (ε_p and ε_i are the permittivities of the particle and medium i, respectively), the magnitude of the dipole terms should be approximately proportional to R_d^6. Furthermore, it is worth noting that, as the incident angle $\theta_I\rightarrow 0°$ or 90°, the magnitude of the dipole moment terms tends to zero, thus leading to a zero LOF. For normal incidence ($\theta_I\rightarrow 0°$), the scattering field is azimuthally symmetric, leading to the disappearance of MSB and LOF. For the grazing incidence ($\theta_I\rightarrow 90°$), the transmission coefficients $t_s(\theta_I)$ and $t_p(\theta_I)$ turn zero, and the dual-dipole scattering interaction vanishes, resulting in a zero LOF.

For analytical evaluation of integral I_1 and I_2 in Eqs. (19-21), we consider two cases of $\varepsilon_{12}>1$ and $\varepsilon_{12}<1$.

a) Incident from optically less dense medium ($\varepsilon_{12}<1$)

For the incident wave from the less dense medium to the denser medium, the integrals I_1 and I_2 have real value over the interval $[0,\infty]$. The integral region is divided into three intervals for discussion when the two dipoles close to ($\xi\rightarrow 0$) or far from (large ξ) the interface between two media.

a.1) For the case of the two dipoles located close to the interface ($\xi\rightarrow 0$)

In the region of integration $[0,\sqrt{\varepsilon_{12}}]$, the transmission coefficient $t_1^p(s)$ and $t_2^p(s)$ are real-valued and the integral can be obtained analytically by direct integration

$$\begin{aligned}&\mathrm{Re}\left[I_1(\varepsilon_{12})\right]\\&=\frac{1}{32(\varepsilon_{12}-1)(\varepsilon_{12}+1)^3}\left\{\begin{array}{l}4(\varepsilon_{12}-1)^2\left(1+6\varepsilon_{12}+\varepsilon_{12}^2\right)arc\,\mathrm{Tanh}\left(\dfrac{\varepsilon_{12}+1}{\varepsilon_{12}-2\sqrt{\varepsilon_{12}}-1}\right)\\+(\varepsilon_{12}+1)\left[4\left(\sqrt{\varepsilon_{12}}-2\varepsilon_{12}^{3/2}-4\varepsilon_{12}^2+\varepsilon_{12}^{5/2}+2\varepsilon_{12}^3+2\varepsilon_{12}^4\right)-(\varepsilon_{12}+1)^3\ln\varepsilon_{12}\right]\end{array}\right\},\ \xi\rightarrow 0,\end{aligned}\tag{27}$$

$$\mathrm{Re}\left[I_2(\varepsilon_{12})\right]=\frac{1}{8(\varepsilon_{12}-1)(\varepsilon_{12}+1)^3}\left\{\begin{array}{l}\varepsilon_{12}^{3/2}\left(2\varepsilon_{12}^{5/2}-\varepsilon_{12}^3+\varepsilon_{12}^2+\varepsilon_{12}-2\sqrt{\varepsilon_{12}}-1\right)\\+\varepsilon_{12}(\varepsilon_{12}-1)^2\left(1+6\varepsilon_{12}+\varepsilon_{12}^2\right)arc\,\mathrm{Tanh}\sqrt{\varepsilon_{12}}+4\varepsilon_{12}^3\ln\varepsilon_{12}\end{array}\right\},\ \xi\rightarrow 0.\tag{28}$$

The expressions of $\mathrm{Re}[I_1(\varepsilon_{12})]$ and $\mathrm{Re}[I_2(\varepsilon_{12})]$ could also be obtained by direct integration over the interval $[\sqrt{\varepsilon_{12}},1]$ where transmission coefficient $t_1^p(s)$ and $t_2^p(s)$ are complex-valued

$$\mathrm{Re}\left[I_1(\varepsilon_{12})\right]=\frac{\varepsilon_{12}\left(1-2\varepsilon_{12}-\varepsilon_{12}^2\right)}{4\left(\varepsilon_{12}+1\right)^2}+\frac{\varepsilon_{12}^2\ln\varepsilon_{12}}{\left(\varepsilon_{12}-1\right)\left(\varepsilon_{12}+1\right)^3}\ ,\ \xi\to 0, \tag{29}$$

$$\mathrm{Re}\left[I_2(\varepsilon_{12})\right]=\frac{1+\varepsilon_{12}\left(3+\varepsilon_{12}-\varepsilon_{12}^2\right)}{4\left(\varepsilon_{12}+1\right)^3}-\frac{\varepsilon_{12}^3\ln\varepsilon_{12}}{\left(\varepsilon_{12}-1\right)\left(\varepsilon_{12}+1\right)^3},\ \xi\to 0. \tag{30}$$

In the region of integration $[1,\infty]$, the integrand of the integrals in Eqs. (19-20) is all real-valued. The largest contribution of the integral arises from the extreme point s_0 of the integrand and its immediate vicinity. In particular, $s_0(\varepsilon_{12},\xi)$ tends to infinity as ξ decreases to zero, and for a given ξ, the real part of integrals will converge on $[1,\infty]$. It should be noted that when $\xi>1$, for integral I_2, the integrand of the integrals has no extreme value, and the maximum value is located at the point $s=1$. Expand the exponential factor $\exp\left[i\xi\left(\sqrt{\varepsilon_{12}\text{-}s^2}+\varepsilon_{12}\sqrt{1\text{-}s^2}\right)\right]$ in series at s_0, and the final expression of Eq. (19) or Eq. (20) can be obtained by directly integrating each term of the series $I_i=\sum_{n=0}^{\infty}\int_1^{\infty}h_i(s)\left[f^{(n)}(s_0)(s-s_0)^n/n!+R_n(s)\right]ds$ (i=1,2). $h_i(s)$ is the non-exponential term of the integrand in I_1 or I_2 and $f^{(n)}(s_0)$ is the n-order derivative of the exponential factor at s_0. Here, the integrals of the first term (n=0) of the series over the interval $[1,2s_0]$ is exhibited,

$$\mathrm{Re}\left[I_1(\varepsilon_{12},\xi)\right]\sim\frac{\exp\left[-\xi\left(\sqrt{s_0^2-\varepsilon_{12}}+\varepsilon_{12}\sqrt{s_0^2-1}\right)\right]}{8\left(\varepsilon_{12}-1\right)\left(\varepsilon_{12}+1\right)^3}\left\{\begin{array}{l}\left(\varepsilon_{12}^2-1\right)\left(\sqrt{AB}\varepsilon_{12}-2\varepsilon_{12}-\sqrt{AB}\right)\\-8s_0^2\left(\varepsilon_{12}+1\right)\left(\sqrt{AB}\varepsilon_{12}+2\varepsilon_{12}+\sqrt{AB}\right)\\+\left(\varepsilon_{12}-1\right)^2\left(\varepsilon_{12}^2+6\varepsilon_{12}+1\right)arc\,\mathrm{Tanh}\left(\sqrt{A/B}\right)\\-8\varepsilon_{12}^2\ln\left(4s_0^2-\sqrt{AB}\right)+32s_0^4\varepsilon_{12}\left(\varepsilon_{12}+1\right)^2\end{array}\right\}, \tag{31}$$

$$\mathrm{Re}\left[I_2(\varepsilon_{12},\xi)\right]\sim\frac{\exp\left[-\xi\left(\sqrt{s_0^2-\varepsilon_{12}}+\varepsilon_{12}\sqrt{s_0^2-1}\right)\right]}{8\left(1-\varepsilon_{12}\right)\left(1+\varepsilon_{12}\right)^3}\left\{\begin{array}{l}\left(\varepsilon_{12}^2-1\right)\left(\sqrt{AB}\varepsilon_{12}^2-\sqrt{AB}\varepsilon_{12}+4\varepsilon_{12}+2\right)\\-8s_0^2\varepsilon_{12}\left(1+\varepsilon_{12}\right)\left(\sqrt{AB}\varepsilon_{12}+2\varepsilon_{12}+\sqrt{AB}\right)\\+\varepsilon_{12}\left(\varepsilon_{12}-1\right)^2\left(\varepsilon_{12}^2+6\varepsilon_{12}+1\right)arc\,\mathrm{Tanh}\left(\sqrt{A/B}\right)\\-8\varepsilon_{12}^3\ln\left(4s_0^2-\sqrt{AB}\right)+32s_0^4\left(1+\varepsilon_{12}\right)^2\end{array}\right\}, \tag{32}$$

here, $A=4s_0^2-1$, $B=4s_0^2-\varepsilon_{12}$.

It is worth noting that one can obtain the expressions of the integral I_1 and I_2 by direct integration in the interval $[1,\infty]$ for the case of high dielectric contrast (ε_{12}<<1).

$$\mathrm{Re}\left[I_1(\xi)\right]\sim\frac{\xi K_1(\xi)+3K_2(\xi)}{\xi^2}\ ,\ \ \varepsilon_{12}\ll 1, \tag{33}$$

$$\mathrm{Re}\left[I_2(\xi)\right]\sim\frac{\xi^3+3\xi^2+6\xi+6}{\xi^4}\exp(-\xi),\ \ \varepsilon_{12}\ll 1, \tag{34}$$

where K_n is the n-order modified Bessel function of the second kind.

Combining Eqs. (25-26) and Eqs. (33-34), the total LOF in Eq. (21) can be expressed as

$$\langle F_y \rangle \sim -\frac{k_0^2 k_2^2}{8\pi\varepsilon_0}\alpha_{e,1}\alpha_{e,2}\left|E_{inc}\right|^2 \sin(2\alpha)\left[\frac{k_2 z_1 K_1(k_2 z_1)+3K_2(k_2 z_1)}{(k_2 z_1)^2}\psi(\theta_I, z_1)\right]. \quad (35)$$

Here, $\psi(\theta_I, z_1) = t_s(\theta_I)\, sin(\theta_I)\left[cos(\delta_{d12}) + r_p(\theta_I)\, cos(\delta_{ref} - \delta_{d12})\right]$. In particular, $K_n(\xi) \sim \frac{1}{2}\Gamma(n)\left(\frac{1}{2}\xi\right)^{-n}$ for the case of $\xi \to 0$[4]. The Eqs. (33-34) can be further simplified as

$$\mathrm{Re}\left[I_1(\xi)\right] = \mathrm{Re}\left[I_2(\xi)\right] \sim \frac{6}{\xi^4}. \quad (36)$$

Therefore, the total LOF can be simplified as follows in the case of $\xi \to 0$

$$\langle F_y \rangle \sim -\frac{3k_0^2 k_2^2}{4\pi\varepsilon_0 (k_2 z_1)^4}\alpha_{e,1}\alpha_{e,2}\left|E_{inc}\right|^2 \sin(2\alpha)\psi(\theta_I, z_1),\ \xi \to 0,\ \varepsilon_{12} \ll 1. \quad (37)$$

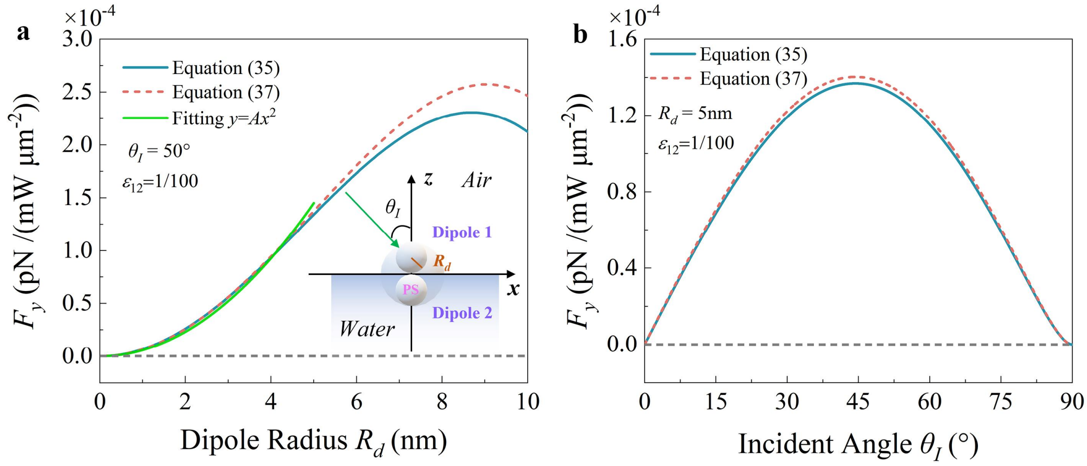

Supplementary Figure 5. (**a**) The normalized LOF in Eqs. (35) and (37) when the dipole radius R_d (dipole separation z_1) ranges from 1 nm to 10 nm and $\varepsilon_{12} = 1/100$, $\theta_I = 50°$. (**b**) The normalized LOF of two dipoles with a radius of 5 nm varies with the incident angle θ_I. The irradiance of the plane wave is set to 1 mW/μm^2.

It is seen that the LOF exhibits a sin(2α) dependence and varies with the incident angle θ_I and the dipole radius R_d ($|z_1|=|z_2|=R_d$). Supplementary Fig. 5a shows the normalized LOF in Eqs. (35) and (37) when the dipole radius R_d (approximately equivalent to dipole separation $|z_1|$) ranges from 1 nm to 10 nm. The dipole model is shown in the inset of Supplementary Fig. 5a. In calculation, $\theta_I = 50°$, $\alpha = 45°$, $\varepsilon_{12} =$ 1/100, the LOF of the two dipoles can be described by the approximate expression of Eq. (37) when $\xi \to 0$. When the $\xi \to 0$, $\alpha_{e,1}\alpha_{e,2} \propto R_d^6$ and $\mathrm{Re}(\partial_y \overleftrightarrow{G}^{tr}_{1,yz}) \propto 1/R_d^4$ leads to the

LOF∝R_d^2 as shown by the quadratic function fitting of the green line in Supplementary Fig. 5a. The high fitted correlation coefficient of 0.99 confirms the R_d^2 way. Supplementary Fig. 5b shows the LOF of two dipoles with a radius of 5 nm as a function of the incident angle and the force shows a gentle change that first increases and then decreases with the incident angle.

a.2) For the case of large ξ ($\xi\to\infty$)

For the case of large ξ, in the interval $[0, \sqrt{\varepsilon_{12}}]$, the asymptotic evaluation of the integral I_1 and I_2 can be performed by the method of stationary phase with stationary point $s_s = 0$[5]. Performing asymptotic evaluation with accuracy up to $1/\xi^2$ term, the $\mathrm{Re}[I_1(\varepsilon_{12},\xi)]$ and $\mathrm{Re}[I_2(\varepsilon_{12},\xi)]$ can be expressed as

$$\mathrm{Re}\left[I_2\left(\varepsilon_{12},\xi\right)\right]=\sqrt{\varepsilon_{12}}\,\mathrm{Re}\left[I_1\left(\varepsilon_{12},\xi\right)\right]\sim-\frac{2\varepsilon_{12}\cos\left[\xi\left(\sqrt{\varepsilon_{12}}+\varepsilon_{12}\right)\right]}{\xi^2\left(1+\sqrt{\varepsilon_{12}}\right)^3\left(1-\sqrt{\varepsilon_{12}}+\varepsilon_{12}\right)^2},\ \xi\to\infty. \tag{38}$$

In the interval $[\sqrt{\varepsilon_{12}}, 1]$, the exponential terms of the integral can be expressed as $\exp\left[-\xi\sqrt{s^2-\varepsilon_{12}}+i\xi\varepsilon_{12}\sqrt{1-s^2}\right]$. One can extract the real part of the integral and perform a Taylor expansion on the trigonometric term at $\sqrt{\varepsilon_{12}}$ since the contribution of the integral comes mainly from the lower limit of the integral. Finally, the integral can be asymptotically expanded by converting them into Laplace form[6] using the replacement of $s=\sqrt{t^2+\varepsilon_{12}}$. Limiting ourselves to the terms up to $1/\xi^3$ we can obtain the following expression:

$$\mathrm{Re}\left[I_1\left(\varepsilon_{12},\xi\right)\right]\sim\frac{\cos\left(\xi\varepsilon_{12}\sqrt{1-\varepsilon_{12}}\right)}{\xi^2}+\frac{\sin\left(\xi\varepsilon_{12}\sqrt{1-\varepsilon_{12}}\right)}{\xi^3}\frac{\varepsilon_{12}^2+2}{2\varepsilon_{12}\sqrt{1-\varepsilon_{12}}},\ \xi\to\infty, \tag{39}$$

$$\mathrm{Re}\left[I_2\left(\varepsilon_{12},\xi\right)\right]\sim-\frac{\sin\left(\xi\varepsilon_{12}\sqrt{1-\varepsilon_{12}}\right)}{\xi^3\sqrt{1-\varepsilon_{12}}},\ \xi\to\infty. \tag{40}$$

In the interval $[1, \infty]$, the integral has a sharp maximum at a point very near the lower limit of the integration, and most of the contribution to the integral arises from the immediate vicinity of this maximum. Therefore, the integral can be evaluated asymptotically by converting them into Laplace form[6] using the replacement of $s=\sqrt{t^2+1}$. Additionally, the Taylor expansion is performed at the lower limit for the decay factor $\exp\left(-\xi\sqrt{s^2-\varepsilon_{12}}\right)$. Limiting ourselves to the terms up to $1/\xi^4$ we can obtain the following expressing

$$\mathrm{Re}\left[I_1(\varepsilon_{12},\xi)\right]\sim\left(\frac{1}{\xi^3\varepsilon_{12}\sqrt{1-\varepsilon_{12}}}-\frac{2\varepsilon_{12}+1}{2\xi^4\varepsilon_{12}(1-\varepsilon_{12})}\right)\exp\left(-\xi\sqrt{1-\varepsilon_{12}}\right),\ \xi\to\infty, \tag{41}$$

$$\mathrm{Re}\left[I_2(\varepsilon_{12},\xi)\right]\sim\left(\frac{1}{\xi^2\varepsilon_{12}}-\frac{2\varepsilon_{12}+1}{2\xi^3\varepsilon_{12}\sqrt{1-\varepsilon_{12}}}+\frac{2\varepsilon_{12}^2-\varepsilon_{12}+2}{2\xi^4\varepsilon_{12}(1-\varepsilon_{12})}\right)\exp\left(-\xi\sqrt{1-\varepsilon_{12}}\right),\ \xi\to\infty. \tag{42}$$

Combining Eqs. (38-42) and leaving only terms to $1/\xi^2$, one can get the final asymptotic evaluation of the derivative of the Green's function:

$$\mathrm{Re}\left(\partial_y\vec{G}_{1,yz}^{tr}\right)\sim\frac{k_2^2}{4\pi}\left(\frac{2\sqrt{\varepsilon_{12}}\cos\left[\xi\left(\sqrt{\varepsilon_{12}}+\varepsilon_{12}\right)\right]}{\xi^2\left(1+\sqrt{\varepsilon_{12}}\right)^3\left(1-\sqrt{\varepsilon_{12}}+\varepsilon_{12}\right)^2}-\frac{\cos\left(\xi\varepsilon_{12}\sqrt{1-\varepsilon_{12}}\right)}{\xi^2}\right),\ \xi\to\infty, \tag{43}$$

$$\mathrm{Re}\left(\partial_y\vec{G}_{2,yz}^{tr}\right)\sim\frac{k_2^2}{4\pi}\left(\frac{2\varepsilon_{12}\cos\left[\xi\left(\sqrt{\varepsilon_{12}}+\varepsilon_{12}\right)\right]}{\xi^2\left(1+\sqrt{\varepsilon_{12}}\right)^3\left(1-\sqrt{\varepsilon_{12}}+\varepsilon_{12}\right)^2}-\frac{\exp\left(-\xi\sqrt{1-\varepsilon_{12}}\right)}{\xi^2\varepsilon_{12}}\right),\ \xi\to\infty. \tag{44}$$

It can be seen from Eqs. (42-43) that the real part of the Green's function includes retarded $\xi\left(\sqrt{\varepsilon_{12}}+\varepsilon_{12}\right)$ and non-oscillating $1/\xi^2$ with $\xi\to\infty$.

b) Incident from optically denser medium ($\varepsilon_{12}>1$)

For the incident wave from the denser medium to the less dense medium, the integrals I_1 and I_2 also have real value over the interval $[0,\infty]$. The integral region is divided into three intervals for discussion when the two dipoles close to ($\xi\to0$) or far from (large ξ) the interface between the two media.

b.1) For the case of the two dipoles located close to the interface ($\xi\to0$)

In the region of $[0, 1]$, the integral can be obtained analytically by direct integration

$$\mathrm{Re}\left[I_1(\varepsilon_{12})\right]=\frac{\sqrt{\varepsilon_{12}}\left(\varepsilon_{12}^2-1\right)\left(\varepsilon_{12}+2\sqrt{\varepsilon_{12}}-1\right)-\left(\varepsilon_{12}-1\right)^2\left(\varepsilon_{12}^2+6\varepsilon_{12}+1\right)arc\mathrm{Coth}\sqrt{\varepsilon_{12}}+4\varepsilon_{12}^2\ln\varepsilon_{12}}{8\left(\varepsilon_{12}-1\right)\left(\varepsilon_{12}+1\right)^3},\ \xi\to0, \tag{45}$$

$$\mathrm{Re}\left[I_2(\varepsilon_{12})\right]=-\frac{1}{8\left(\varepsilon_{12}-1\right)\left(\varepsilon_{12}+1\right)^3}\left\{\begin{aligned}&2\left(\varepsilon_{12}^2-1\right)\left(\varepsilon_{12}^{5/2}-\varepsilon_{12}^{3/2}-4\varepsilon_{12}-2\right)+8\varepsilon_{12}^3\ln\varepsilon_{12}\\&+\varepsilon_{12}\left(\varepsilon_{12}-1\right)\left[\ln\left(\frac{\sqrt{\varepsilon_{12}}+1}{\sqrt{\varepsilon_{12}}-1}\right)+\left(\varepsilon_{12}^3+5\varepsilon_{12}^2-5\varepsilon_{12}\right)\ln\left(\frac{\sqrt{\varepsilon_{12}}-1}{\sqrt{\varepsilon_{12}}+1}\right)\right]\end{aligned}\right\},\ \xi\to0. \tag{46}$$

In the region of $[1,\sqrt{\varepsilon_{12}}]$, the transmission coefficient is complex-valued, the real part of the derivative of Green's functions can be obtained analytically by direct integration,

$$\mathrm{Re}\left[I_1(\varepsilon_{12})\right]=\frac{\varepsilon_{12}\left(\varepsilon_{12}^2+2\varepsilon_{12}-1\right)}{4\left(\varepsilon_{12}+1\right)^2}-\frac{\varepsilon_{12}^2\ln\varepsilon_{12}}{\left(\varepsilon_{12}-1\right)\left(\varepsilon_{12}+1\right)^3},\ \xi\to0, \tag{47}$$

$$\mathrm{Re}\left[I_2(\varepsilon_{12})\right]=\frac{\varepsilon_{12}^2-2\varepsilon_{12}-1}{4\left(\varepsilon_{12}+1\right)^2}+\frac{\varepsilon_{12}^3\ln\varepsilon_{12}}{\left(\varepsilon_{12}-1\right)\left(\varepsilon_{12}+1\right)^3},\ \xi\to0. \tag{48}$$

In the region of $[\sqrt{\varepsilon_{12}}, \infty]$, similar to the case of integration in the interval $[1, \infty]$ when $\varepsilon_{12}<1$, the following expression can be obtained

$$\mathrm{Re}\left[I_1(\varepsilon_{12},\xi)\right] \sim \frac{\exp\left[-\xi\left(\sqrt{s_0^2-\varepsilon_{12}}+\varepsilon_{12}\sqrt{s_0^2-1}\right)\right]}{8(\varepsilon_{12}-1)(\varepsilon_{12}+1)^3}\left\{\begin{matrix}(\varepsilon_{12}-1)^2(\varepsilon_{12}^2+6\varepsilon_{12}+1)arc\mathrm{Tanh}\left(\sqrt{B/A}\right)\\ -8s_0^2(\varepsilon_{12}+1)\left(\sqrt{AB}\varepsilon_{12}+2\varepsilon_{12}+\sqrt{AB}\right)\\ -\left(\varepsilon_{12}^2-1\right)\left(2\varepsilon_{12}^3+4\varepsilon_{12}^2-\sqrt{AB}\varepsilon_{12}+\sqrt{AB}\right)\\ +8\varepsilon_{12}^2\ln\left(\dfrac{\varepsilon_{12}}{4s_0^2-\sqrt{AB}}\right)+32s_0^4\varepsilon_{12}(\varepsilon_{12}+1)^2\end{matrix}\right\}, \tag{49}$$

$$\mathrm{Re}\left[I_2(\varepsilon_{12},\xi)\right] \sim \frac{\exp\left[-\xi\left(\sqrt{s_0^2-\varepsilon_{12}}+\varepsilon_{12}\sqrt{s_0^2-1}\right)\right]}{8(1-\varepsilon_{12})(1+\varepsilon_{12})^3}\left\{\begin{matrix}\varepsilon_{12}(\varepsilon_{12}-1)^2(\varepsilon_{12}^2+6\varepsilon_{12}+1)arc\mathrm{Tanh}\left(\sqrt{B/A}\right)\\ -8s_0^2\varepsilon_{12}(1+\varepsilon_{12})\left(\sqrt{AB}\varepsilon_{12}+2\varepsilon_{12}+\sqrt{AB}\right)\\ +\varepsilon_{12}\left(\varepsilon_{12}^2-1\right)\left(\sqrt{AB}\varepsilon_{12}+2\varepsilon_{12}-\sqrt{AB}\right)\\ +8\varepsilon_{12}^3\ln\left(\dfrac{\varepsilon_{12}}{4s_0^2-\sqrt{AB}}\right)+32s_0^4(1+\varepsilon_{12})^2\end{matrix}\right\}, \tag{50}$$

b.2) For the case of large ξ ($\xi\to\infty$)

For the case of large ξ, the integral in Eqs. (19-20) can also be evaluated asymptotically by the method of stationary phase with stationary point $s_s = 0$ in the interval $[0, 1]$. The integral result is the same as Eq. (38).

For the interval $[1, \sqrt{\varepsilon_{12}}]$, the exponential terms of the integral I_1 and I_2 could be simplified as $\exp\left(i\xi\sqrt{\varepsilon_{12}-s^2}\right)$ and the integral I_1 and I_2 can be obtained by the method of stationary phase[5]. Using the substitution of integration variable $s=\sqrt{t^2+1}$ to avoid divergences in the endpoints, one can get

$$\mathrm{Re}\left[I_1(\varepsilon_{12},\xi)\right] \sim -\frac{\sin\left(\xi\sqrt{\varepsilon_{12}-1}\right)}{\xi^3\varepsilon_{12}\sqrt{\varepsilon_{12}-1}},\ \xi\to\infty, \tag{51}$$

$$\mathrm{Re}\left[I_2(\varepsilon_{12},\xi)\right] \sim \frac{\cos\left(\xi\sqrt{\varepsilon_{12}-1}\right)}{\varepsilon_{12}\xi^2}+\frac{\sin\left(\xi\sqrt{\varepsilon_{12}-1}\right)}{\xi^3}\frac{2\varepsilon_{12}+1}{2\varepsilon_{12}\sqrt{\varepsilon_{12}-1}},\ \xi\to\infty. \tag{52}$$

In the interval $[\sqrt{\varepsilon_{12}}, \infty]$, the integral has a sharp maximum at a point very near the lower limit of the integration, and most of the contribution to the integral arises from the immediate vicinity of this maximum. Therefore, the integral can be evaluated asymptotically by converting them into Laplace form[6] using the replacement of $s=\sqrt{t^2+\varepsilon_{12}}$. Additionally, the Taylor expansion is performed at the lower limit for the decay factor $\exp\left(-\xi\varepsilon_{12}\sqrt{s^2-1}\right)$. Limiting ourselves to the terms up to $1/\xi^4$ we can obtain the following expressing

$$\mathrm{Re}\left[I_1(\varepsilon_{12},\xi)\right]\sim\left(\frac{1}{\xi^2}-\frac{\varepsilon_{12}^2+2}{2\xi^3\varepsilon_{12}\sqrt{\varepsilon_{12}-1}}+\frac{3\varepsilon_{12}^2-2\varepsilon_{12}+2}{2\xi^4\varepsilon_{12}^2(\varepsilon_{12}-1)}\right)\exp\left(-\xi\varepsilon_{12}\sqrt{\varepsilon_{12}-1}\right),\ \xi\to\infty, \quad (53)$$

$$\mathrm{Re}\left[I_2(\varepsilon_{12},\xi)\right]\sim\left(\frac{1}{\xi^3\sqrt{\varepsilon_{12}-1}}-\frac{\varepsilon_{12}^2+2}{2\xi^4\varepsilon_{12}(\varepsilon_{12}-1)}\right)\exp\left(-\xi\varepsilon_{12}\sqrt{\varepsilon_{12}-1}\right),\ \xi\to\infty. \quad (54)$$

Supplementary Note 5: Lateral mirror symmetry breaking of spherical Rayleigh particles.

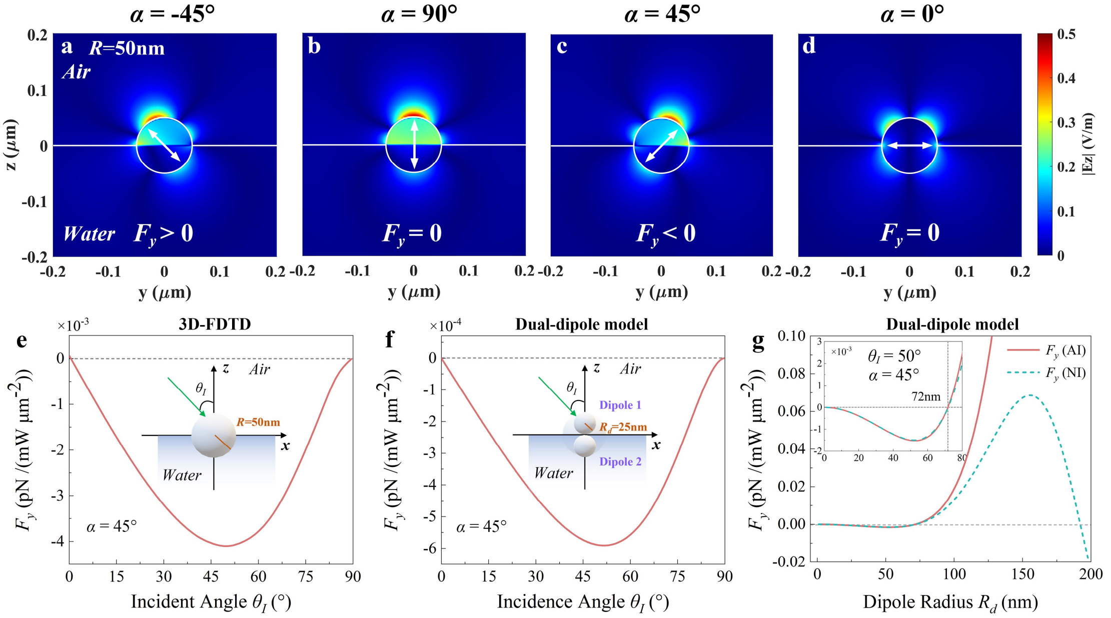

Supplementary Figure 6. (**a-d**) Scattering field $|E_z|$ in the yz-plane for a 50 nm radius PS particle under different polarization orientation α, where $\theta_I = 50°$. (**e**) The LOF of a particle with a radius of 50 nm was calculated by the 3D-FDTD method, where $\alpha = 45°$. (**f**) The LOF of two particles with a radius of 25 nm was calculated by the dual-dipole model, where $\alpha = 45°$. (**g**) Dependence of the LOF on the radius calculated by the numerical integration (NI) and asymptotic integration (AI) in dual-dipole theory, where $\alpha = 45°$, $\theta_I = 50°$. The irradiance of the plane wave in (**f**) and (**g**) is set to 1 mW/μm^2.

To verify the validity of the proposed dual-dipole model, the 3D-FDTD method was used to numerically simulate the scattering near-field and calculate the LOF on a 50 nm radius PS particle, as shown in Supplementary Fig. 6. The nanoparticle was placed at the interface of water and air and illuminated with an LP plane wave of $\theta_I = 50°$.

Supplementary Figs. 6a-6d shows the scattering near-field $|E_z|$ in the yz-plane for a PS particle with a 50nm radius at different polarization angles α. It is seen that for the polarization orientation $\alpha = 0°$ (Supplementary Fig. 6d) and 90° (Supplementary Fig. 6b), the scattering near-field $|E_z|$ presents a mirror-symmetric distribution, while for the polarization orientation $\alpha = -45°$ (Supplementary Fig. 6a) and 45° (Supplementary Fig. 6c), the mirror symmetry of the scattering near-field is broken and thus leading to an

LOF acting on the particle. In particular, for α = -45° (45°), the scattering near-field of 50 nm radius Rayleigh particle is mainly concentrated in the $-y$ ($+y$) direction, which is opposite to that of 500 nm radius Mie PS particle (see Fig. 2b and Fig. 2d in the main text). This indicates that at the same polarization angle, the two sizes of particles have LOFs with opposite signs. When the polarization angle α = 45°, the dependence of LOF calculated by 3D-FDTD on incident angle θ_I is shown in Supplementary Fig. 6e. The inset is a schematic diagram of the simulation model. In Supplementary Fig. 6e, the LOF gradually increases to the maximum as the incident angle θ_I increases to 50° and then decreases to zero as the incident angle increases. Supplementary Fig. 6f shows the LOF calculated by numerical integration (NI) of dual-dipole theory. The inset is the theoretical model of the dual-dipole theory, two spherical dipoles with a radius of 25 nm are equivalent to a PS particle with a radius of 50 nm. The dependence of LOF on the incident angle θ_I calculated by the dual-dipole theory has the same variation trend as that obtained by 3D-FDTD method. It is verified that the proposed dual-dipole model can describe the MSB mechanism induced by a single LP plane wave under dipole approximation. Additionally, the deviation of LOF between the asymptotic integration (AI, $\xi \rightarrow 0$) and the NI was evaluated, as shown in Supplementary Fig. 6g, where θ_I = 50°, α = 45°. The results show that the relative error between AI and NI is less than 2% when the radius of a single spherical dipole is less than 70 nm. This means that when the dipole radius is smaller than 70 nm, LOF can be evaluated with high accuracy by AI of Eq. (19) and Eq. (20). Meanwhile, the NI calculation results also show that when a dipole radius is less than 72 nm, a negative LOF will be generated by an LP light with α > 0°. This also explains why the LOF of Rayleigh particles and Mie particles is opposite at the same polarization angle.

Supplementary Note 6: LOF of a large-scale particle floating at the air-water interface was calculated by using 3D-Raytracing.

The theoretical analysis results discussed above are derived from the dual-dipole model. However, the particle sizes used in the experiments are in the ray optics range (R>10λ, λ = 532 nm). Therefore, we utilize the 3D-Raytracing method to demonstrate that, under the ray optics range, semi-floating spherical particles can nevertheless be exposed to the LOF induced by a single LP wave.

In the 3D-Raytracing method, the incident light comprises an N×N array of rays, each carrying the same initial power to simulate a plane wave. Here, N is set to 1200,

corresponding to a ray spacing of 25 nm, which provides good convergence while reducing computation time. We use vector Snell's law and Fresnel's formula to calculate the direction and intensity of reflected and refracted rays when the refractive index is abruptly changed. It is worth noting that the power carried by a single ray is decomposed into *s*- and *p*-polarization in each refraction and reflection process to calculate the reflected and refracted power. The momentum of a single ray can be expressed by Minkowski momentum $p_M = n\varphi/c$, where n is the refractive index of the medium, φ is the ray power, and c is the speed of light in vacuum. The optical force can be calculated from the change in the momentum of the light per unit time. Therefore, by integrating the momentum changes of all single rays on the particle per unit time, the total momentum change per unit time can be determined, and thus the LOF exerting on the particle can be obtained.

a) The LOF of a spherical PS particle

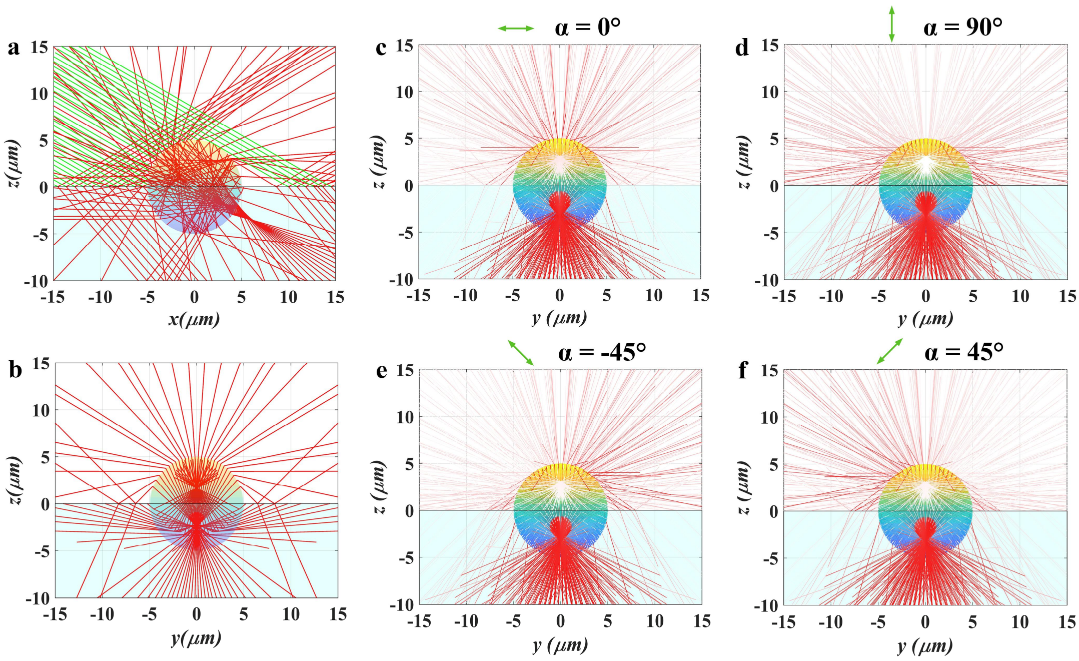

Supplementary Figure 7. 3D-Raytracing patterns. A PS particle with a radius of 5 μm is semi-floating at the air–water interface. (**a-b**) Ray trajectories of low ray density near a PS particle of radius 5 μm in *xz* and *yz* sections, $\theta_I = 60°$. (**c-f**) The 3D rays' normalized intensity distribution in the *yz* perspective when $\alpha = 0°$, 90°, -45° and 45°, respectively.

A PS particle with a radius of 5 μm ($n_p = 1.5983$ @ $\lambda = 532$nm) is half in air ($n_1 = 1$) and half in water ($n_2 = 1.337$), as shown in Supplementary Fig. 7. Supplementary Fig. 7a-7b show the ray trajectories of low ray density for *xz*-plane and *yz*-plane, respectively, where the green rays are the incident light with incident angle of $\theta_I = 60°$. Supplementary Figs. 7c-7f shows the direction and normalized intensity of scattered

rays in the *yz* view at different polarization angles *α*, respectively, and the green arrow corresponds to the direction of electric field vibration. Notably, the normalized intensity of the reflected rays has tripled for ease of viewing. For *α* = 0° or 90°, the ray's intensity in air and water is mirror-symmetric with respect to the *xz*-plane, as shown in Supplementary Fig. 7c and Fig. 7d. However, for *α* = -45° (*α* = 45°), the scattered energy in the air is more concentrated in the +*y* (-*y*) direction, and thus the particle will experience an LOF in the -*y* (+*y*) direction, as shown in Supplementary Fig. 7e and Fig. 7f.

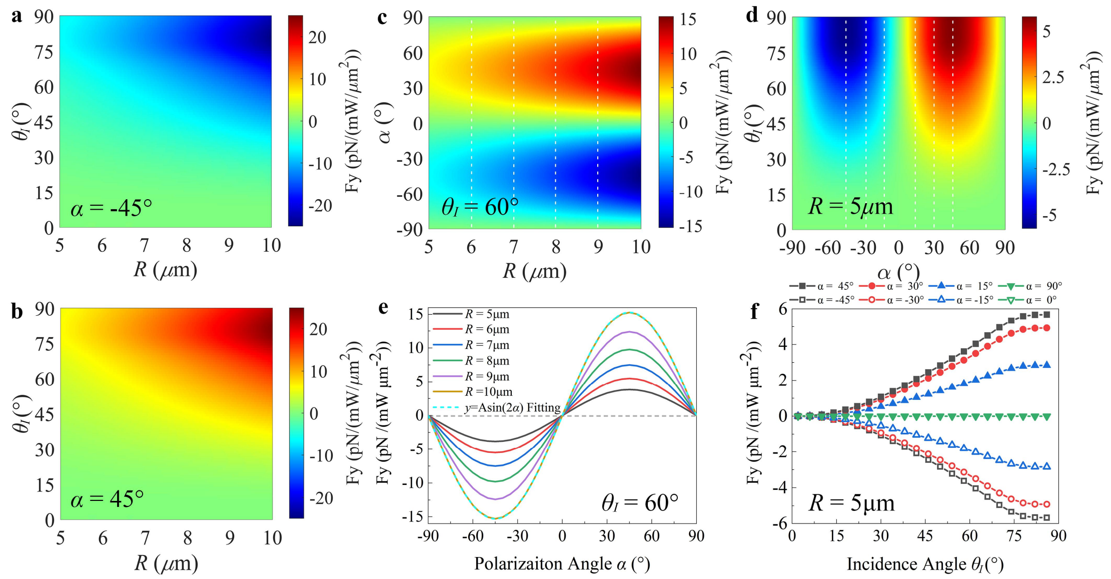

Supplementary Figure 8. Numerical calculation of LOF acting on a PS particle. (**a**) and (**b**) are the dependence of the LOF on the radius *R* and the incident angle θ_I at the polarization angles *α* = -45° and *α* = 45°, respectively. (**c**) The dependence of the LOF on the radius *R* and the polarization angle *α*, where the incident angle θ_I = 60°. (**d**) The dependence of the LOF on the polarization angle *α* and the incident angle θ_I for a 5 μm radius particle. (**e**) Dependence of the LOF on the polarization angle *α* for PS particles with different radii. (**f**) Dependence of the LOF on the incident angle θ_I at different polarization angles *α*.

Relating the momenta of the incident, reflected and transmitted rays in Minkowski formalism, the force exerted on a particle is equal to the change in momentum per unit time, $\Delta \mathbf{p} = -[(\mathbf{p_r} + \mathbf{p_t}) - \mathbf{p_i}]$. Therefore, the LOF can be calculated by integrating $\Delta \mathbf{p}$, as shown in Supplementary Fig. 8. Supplementary Fig. 8a and Fig. 8b show the dependence of the LOF on the particle radius *R* and the incident angle θ_I when the polarization angles *α* = -45° and *α* = 45°, respectively. It is seen that the direction of the LOF is opposite at the polarization angles *α* = -45° and *α* = 45°, and the magnitude of the force increases with the incident angle θ_I or particle radius *R*. Supplementary Fig. 8c shows the dependence of the LOF on the radius *R* and the polarization angle *α* when the incident angle θ_I = 60°. To intuitively show the change of the LOF with the

polarization angle α and the radius R, we extracted the data (white dashed line) at R = 5 μm, 6 μm, 7 μm, 8 μm, 9 μm, and 10 μm from Supplementary Fig. 8c, and shown in Supplementary Fig. 8e. In Supplementary Fig. 8e, the function $y=A\sin(2\alpha)$ is used to fit the LOF (R = 10μm) to verify the functional relationship between LOF and polarization angle α. The fitting result is shown in the cyan dotted line in Supplementary Fig. 8e, and the fitting correlation coefficient is 1. It shows that the dependence of the LOF with the polarization angle α is consistent with that derived from the dual-dipole theory, $F_y \propto sin(2\alpha)$. Supplementary Fig. 8d shows the dependence of the LOF of the 5 μm radius particle on the polarization angle α and the incident angle θ_I. To observe the change of LOF with the incident angle θ_I, we extracted data (white dashed lines) for different polarization angles α in Supplementary Fig. 8d and plotted in Supplementary Fig. 8f. It is seen that as the incident angle θ_I increases, the LOF increases gradually and reaches the maximum at θ_I = 90°. Interestingly, this result is inconsistent with the 3D-FDTD simulation results and the results derived from the dual-dipole theory (θ_I = 90°, F_y=0). This is because in the 3D-Raytracing method, the phase between the rays is not considered, and the incident rays are incoherent light, while in the 3D-FDTD method and dual-dipole theory, the incident light is coherent. For incoherent light, if θ_I = 90°, the largest degree of MSB will be induced at α = 45° (see Fig. 2h in the main text) and thus lead to the maximum LOF.

b) The LOF of lens-like particles

In the experiments, we demonstrate the LOF induced by a single LP wave using dodecane oil droplets (n_p = 1.4216 @ λ=532 nm). The oil droplet will transform into a liquid lens when it is released on the liquid surface, as shown in Supplementary Fig. 9a. It is determined by the balance of interfacial tensions at the three-phase contact line of a droplet on the liquid surface[7]. The surface tension of dodecane is γ_d = 25 mN/m, the surface tension of water is γ_w = 71.72 mN/m, and the dodecane–water interfacial tension is γ_{dw} = 51.63 mN/m at the experimental temperature of 25°[7]. By calculating the Neumann triangle[7,8], we obtained the contact angle between the liquid lens and the liquid surface of φ_1 = 30.08°, φ_2 = 14.05°. In addition, the characteristic capillary lengths of air, water and dodecane are greater than 1 mm, which is significantly longer than the ~20 μm diameter oil droplets utilized in the experiment. Therefore, the liquid lens can be regarded as a combination of two spherical caps[9], the shape of which is uniquely determined by the circular three-phase contact line radius R and the contact angle φ.

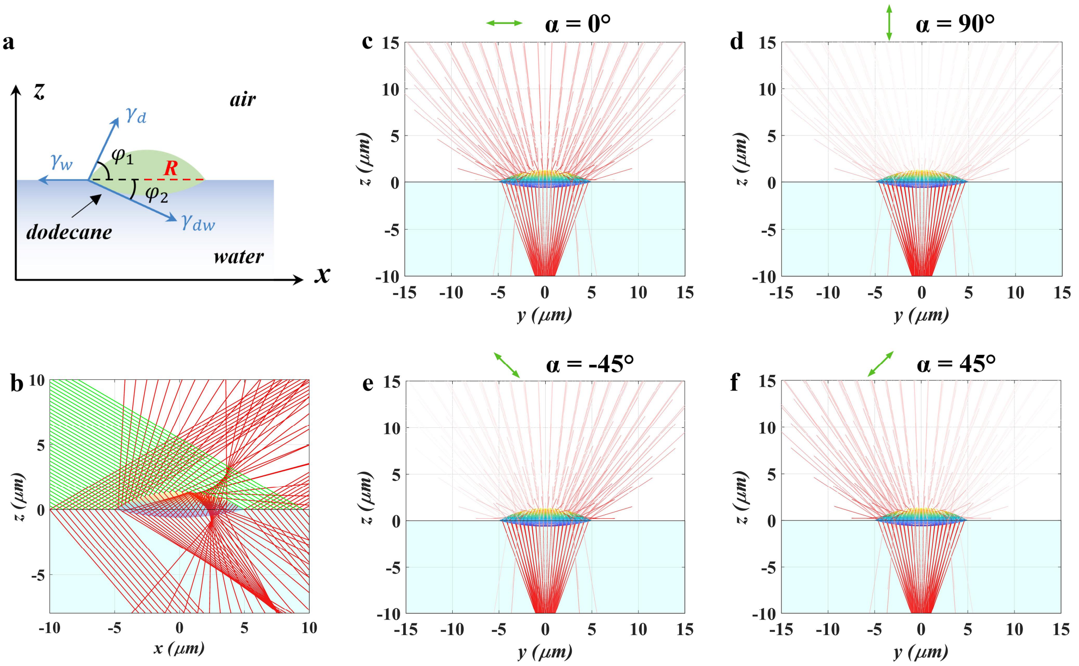

Supplementary Figure 9. 3D-Raytracing patterns. A dodecane oil droplet with a three-phase contact line radius of 5 μm floating at the air-water interface. (**a**) The liquid lens composed of two spherical caps is located on the liquid surface, and the corresponding contact angles are $\varphi_1 = 30.08°$ and $\varphi_2 = 14.05°$, respectively. (**b**) Ray trajectories of low ray density near a 5 μm radius oil droplet in *xz* section, $\theta_I = 60°$. (**c-f**) The 3D rays' normalized intensity distribution in the *yz* perspective when $\alpha = 0°$, 90°, -45° and 45°, respectively.

We established a 3D oil drop model and show the ray trajectories with low ray density in the *xz* section in Supplementary Fig. 9b, with the incident angle of $\theta_I = 60°$, the circular three-phase contact line radius $R = 5$ μm. The directions and normalized intensities of scattered rays in the *yz* view at different polarization angles α are respectively shown in Supplementary Figs. 9c-9f. It is seen that the equivalent focal length of the oil droplet is longer than that of standard spherical PS particles due to the smaller refractive index and surface curvature of the oil droplet. Therefore, the beam can preserve focusing properties over longer propagation distances underwater. For $\alpha = 0°$ or 90°, the ray's intensity in both air and water is mirror-symmetric with respect to the *xz*-plane, as shown in Supplementary Fig. 9c and Fig. 9d. However, for $\alpha = -45°$ (45°), the scattered energy in the air is mainly concentrated in the +*y* (-*y*) direction, thus leading to an LOF in the -*y* (+*y*) direction, as shown in Supplementary Fig. 9e (Fig. 9f). It indicates that the LOF on the oil droplet can also be induced by an LP plane wave.

The dependence of the LOF exerting on the oil droplet on the α, θ_I, and R is estimated, as shown in Supplementary Fig. 10. Supplementary Fig. 10a and Fig. 10b show the dependence of the LOF on the contact line radius R and the incident angle θ_I when the polarization angles $\alpha = -45°$ and $\alpha = 45°$, respectively. It is seen that the direction of the

LOF is opposite at the polarization angles α = -45°and α = 45°, and the magnitude of the force increases with the incident angle θ_I and oil droplet contact line radius R. Supplementary Fig. 10c shows the dependence of the LOF on the R and the α of the oil droplet at θ_I = 60°. To facilitate observation, we extracted data from Supplementary Fig. 10c for R = 5 μm, 6 μm, 7 μm, 8 μm, 9 μm and 10 μm (white dashed lines) to demonstrate the variation of LOF with α, as shown in Supplementary Fig. 10e. In Supplementary Fig. 10e, the dependence of LOF on α is consistent with that of spherical PS particles in Supplementary Fig. 8e, which is proportional to sin(2α), as shown by the fitted curve in cyan. Supplementary Fig. 10d shows that the LOF of the 5 μm radius oil droplet depends on the α and the θ_I. The curve in Supplementary Fig. 10f obtained from Supplementary Fig. 10d represents the variation of the LOF with the incident angle. In Supplementary Fig. 10f, as the incident angle increases, the LOF increases gradually, reaches a maximum at θ_I = 50°, and then decreases with the incident angle. This is due to the fact that as the incident angle θ_I increases and is greater than 50°, the lens-like oil droplet's contact area with the light shrinks, resulting in a reduced scattering cross section and LOF decrease.

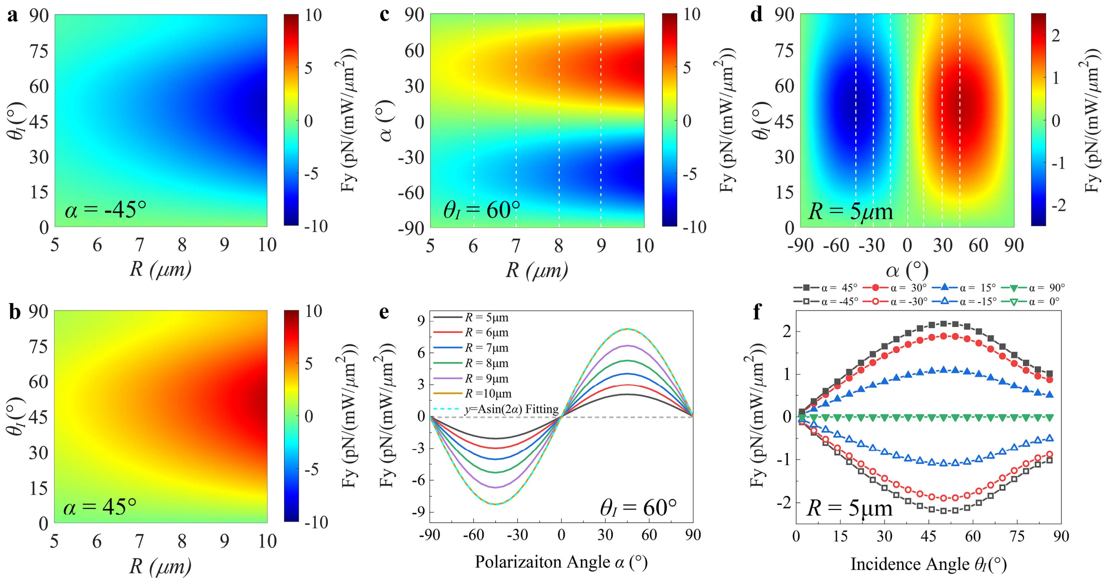

Supplementary Figure 10. Numerical calculation of LOF acting on a dodecane oil droplet. (**a-b**) The dependence of the LOF on the contact line radius R and the incident angle θ_I at the polarization angles α = -45° and α = 45°, respectively. (**c**) The dependence of the LOF on the contact line radius R and the polarization angle α at the incident angle θ_I = 60°. (**d**) The dependence of the LOF on the polarization angle α and the incident angle θ_I for a 5 μm radius droplet. (**e**) Dependence of the LOF on the polarization angle α for droplets with different radii. (**f**) Dependence of the LOF on the incident angle θ_I at different polarization angles α.

From the numerical calculation results in Supplementary Figs. 7-10, it is seen that whether for a spherical PS particle or a lenticular oil droplet located at the air-water

interface, a single LP plane wave can be used to induce MSB and generate LOF. The magnitude and sign of the LOF can be robustly tuned by the incident angle θ_I and polarization angle α. In addition, the LOF is proportional to sin(2α), which is consistent with the dual-dipole theory and numerical simulation results by 3D-FDTD. This further proves that the proposed MSB mechanism applies not only to Rayleigh particles (R<<λ) and Mie particles (R~λ), but also to particles with a radius much larger than the wavelength (R>10λ), making it possible to observe experimentally. Therefore, the proposed MSB mechanism is applicable to $SO(2)$ rotationally symmetric systems of arbitrary size and shape. Interestingly, the LOF obtained in the 3D-Raytracing method does not consider the phase between the rays. This suggests that incoherent light could also induce MSB and generate an observable LOF.

Supplementary Note 7: LOF on Mie lenticular oil droplet floating at the air-water interface.

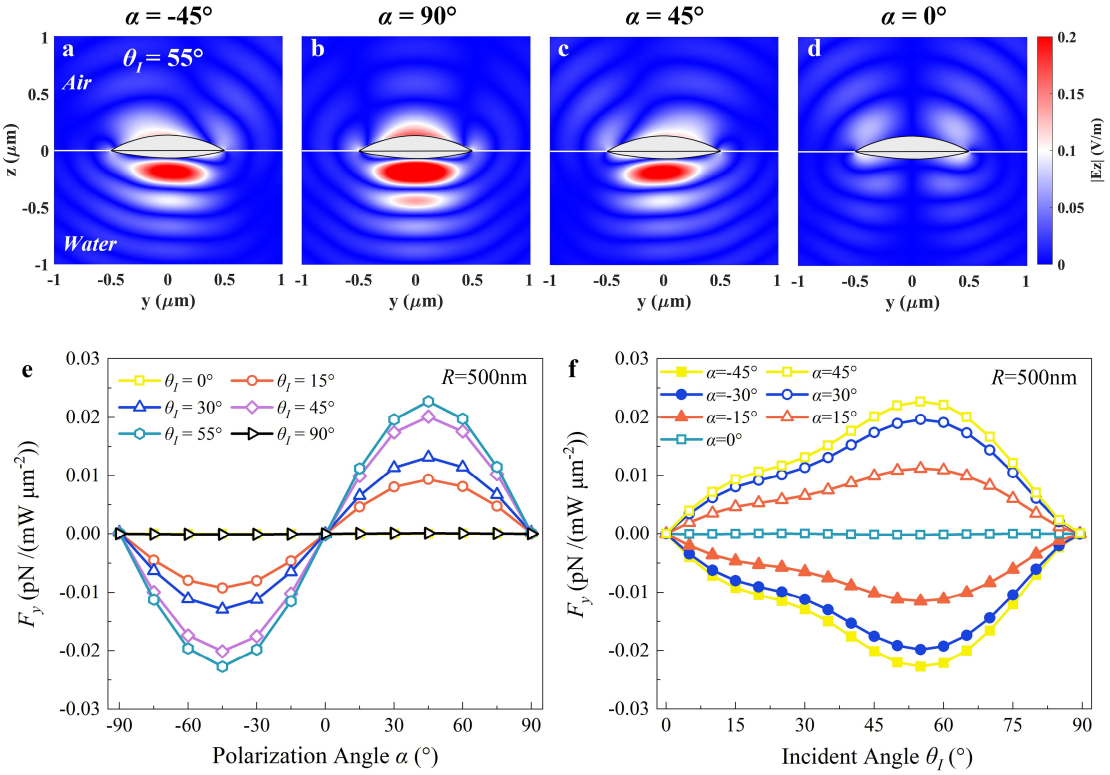

Supplementary Figure 11. Numerical calculation of near-field scattering field and LOF of Mie oil droplets floating at the air-water interface. (**a-d**) Scattering field $|E_z|$ in the yz-plane for a three-phase contact line radius R = 500 nm oil droplet under different polarization angle α. The incident angle is 55°. (**e**) Dependence of the LOF on the polarization angle α at different incident angles θ_I. (**f**) Dependence of the LOF on the incident angle at different polarization angles.

The scattering near-field and LOF of a Mie oil droplet floating at the air-water interface are given, as shown in Supplementary Fig. 11. Supplementary Figs. 11a-11d

show the scattering near-field $|E_z|$ in the yz-plane for an oil droplet with a three-phase contact line radius R = 500 nm at different polarization angles α. The incident angle is 55°. It is seen that for the polarization angle α = 90° (Supplementary Fig. 11b) and 0° (Supplementary Fig. 11d), the scattering near-field $|E_z|$ presents a mirror-symmetric distribution, while for the polarization angle α = -45° (Supplementary Fig. 11a) and 45° (Supplementary Fig. 11c), the mirror symmetry of the scattering field is broken and thus leading to an LOF acting on the oil droplet. The dependence of the LOF on the polarization angle α at different incident angles θ_I is shown in Supplementary Fig. 11e. It is seen that the LOF shows a sin(2α) relationship with the polarization angle α, which is consistent with the calculation results of large-size oil droplets in Supplementary Fig. 10e. This once again proves that an LP plane wave can induce the MSB of light scattering of rotationally symmetric objects of arbitrary size and generation polarization-dependent LOF. In addition, the dependence of the LOF on the incident angle θ_I at different polarization angles α is shown in Supplementary Fig. 11f. As the incident angle increases, the LOF first increases and then decreases. This result is also similar to the LOF of large-sized oil droplets in Supplementary Fig. 10f as a function of incident angle.

Supplementary Note 8: Experimental setups.

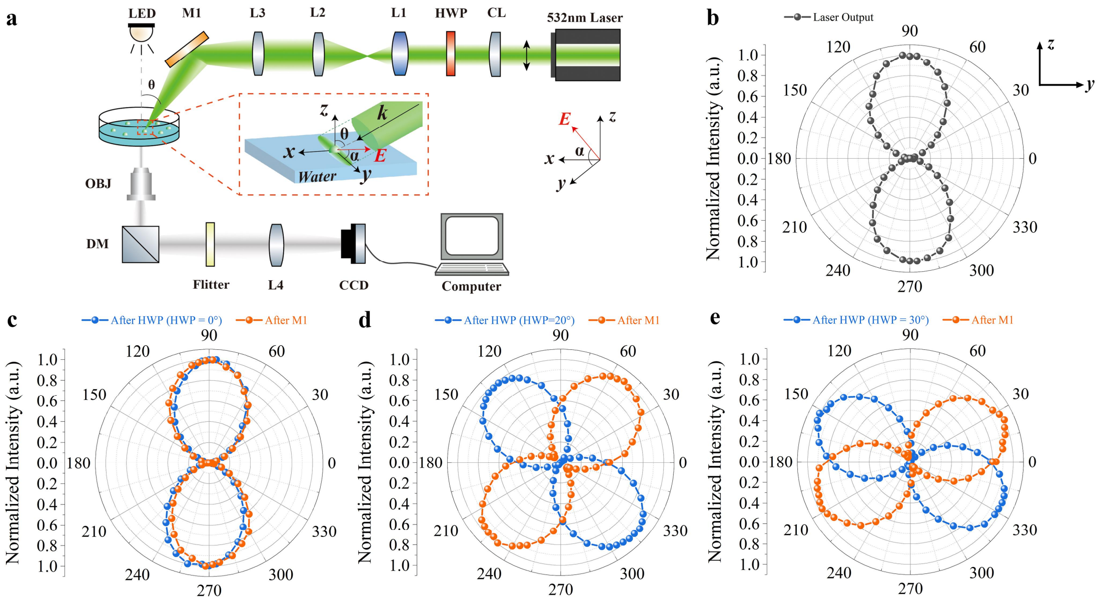

Supplementary Figure 12. Schematic diagram of the experimental set-up and the polarization states of an incident wave at different positions. (**a**) Experimental setup. M1 mirror, CL cylindrical lens (100 mm), HWP half-wave plate (532 nm), L1 lens 1 (50 mm), L2 lens 2 and L3 lens 3 (250 mm), OBJ 10× objective, DM dichroic mirror, L4 lens 4 (200 mm). (**b**) The polarization state of the laser output. (**c-e**) The polarization state of the beam before (blue dotted line) and after (orange dotted line) M1 at the fast axis angle of 0°, 20°, and 30°, respectively.

The experimental setup is shown in Supplementary Fig. 12a. An LP monochromatic CW laser (λ = 532 nm, 1.83 W, Changchun New Industries Optoelectronics Tech. Co., Ltd.) was used to verify the proposed MSB mechanism. The linear polarization state of the Laser output is *p*-polarization, as shown in Supplementary Fig. 12b, and the corresponding extinction ratio is 26.1 dB.

The beam was focused on the liquid surface into a linear trap (dimensions of 100 μm × 900 μm) using a CL with a focal length of 100 mm and a collimator-beam expander lens set. Here, the CL could be rotated precisely to make the linear trap along the y direction to ensure experimentally that the oil droplets only move along the y direction instead of the x direction when illuminated by the beam, as shown in the inset of Supplementary Fig. 12a. The set of the collimator-beam expander lens consists of three positive lenses L1, L2 and L3, of which the focal length of L1 is 50 mm, and the focal lengths of L2 and L3 are 250 mm. The half-wave plate (HWP) is rotated to change the linear polarization direction of the beam, but not change the linear shape of the beam. Accordingly, the polarization direction is governed by a fast axis angle between the HWP fast axis and the $+y$ direction. Supplementary Figs. 12c-12e shows the linear polarization states before (blue dotted line) and after (orange dotted line) M1 when the fast axis angle of the HWP is 0°, 20° and 30°, respectively. It should be noted that all angles start from the $+y$ direction. When the relationship between the fast axis angle of the HWP and the polarization angle α after M1 is calibrated in our experiments, the linear polarization direction of the incident light acting on the sample can be determined indirectly by the HWP fast axis angle. To facilitate the observation of the movement of the oil droplets, the sample was prepared in a transparent glass vessel (~5.5 cm×5.5 cm ×8 mm), and a white LED illuminated the sample from above the liquid surface. A 10× objective lens with a long working distance (Nikon, NA = 0.11) was placed under the sample for imaging, and a CCD with a frame rate of 16 was connected to the computer to record the movement of the oil droplets in real-time. An edge-pass filter (Thorlabs FESH0500) was used to remove scattered light so that the movement of the oil droplets could be clearly recorded by the CCD.

In addition, the system is placed on an optical shock-absorbing platform to reduce external vibrations. A semi-enclosed transparent box encloses the sample, with holes only on one side of the incident light to avoid airflow-induced movement of oil droplets.

Supplementary Note 9: LOF efficiency.

Supplementary Table 1 Experimental comparison of the LOF efficiency of a chiral or achiral particle through different MSB mechanisms.

Material	Mechanisms	Incident wave	Radius (μm)	Beam Size (μm)	Efficiency (pN mW^{-1} μm^{2})	Reference
Chiral	Lateral momenta transfer	LP	1	80×600	0.063	Ref. 10
	Optical helicity gradients	LP	30	~70×2400	79.22	Ref. 11
Achiral	Transverse Belinfante spin momentum	CP	0.77	~10×10	0.035*	Ref. 12
		CP	2.5	~50×1800	0.09	Ref. 13
	Spin-orbit coupling	CP	2.25	30×550	0.016	Ref. 3
	Dipole interaction	LP	10.5	100×900	9.22	This work

Note: LP linearly polarized light, CP circularly polarized light. The force efficiency (pN mW^{-1} μm^{2}) is defined as the LOF (pN) divided by the laser intensity (mW/μm^{2}). The intensity is calculated based on the beam parameters and laser power provided in the reference.

Supplementary Note 10: Supplementary video files.

Media File 1: The video shows the back-and-forth motion of a dodecane oil droplet with a radius R = 9.7 μm in a linear trap when the incident light is switched between polarization angles α = -45° and α = 45°. The green arrow is the projection of the incident light on the xy-plane, along the $+x$ direction. The oil droplet moves along the $+y$ direction when α = 45°, and moves along the $-y$ direction when α = -45°. The speed of the movie is 10 times faster.

Media File 2: The video shows the lateral motion of a dodecane oil droplet with a radius R = 10.5 μm in a linear trap when the incident light is switched between different polarization angles. The speed of the movie is 5 times faster. Extracting the lateral displacement and velocity of the oil droplet at different polarization angles frame by frame is shown in Supplementary Fig. 13. The video and Supplementary Fig. 13 show that movement speed could achieve maximum at α = ±45°, confirming the tunability of the polarization.

Media File 3: The video shows the movement of an aggregate composed of multiple oil droplets in a linear trap when the incident light is switched between polarization angles α = -45° and α = 45°. Over time, multiple oil droplets are captured in the linear trap and move simultaneously. The movement direction of the oil droplets obviously depends on the polarization direction of the LP light. The speed of the movie is 10 times faster.

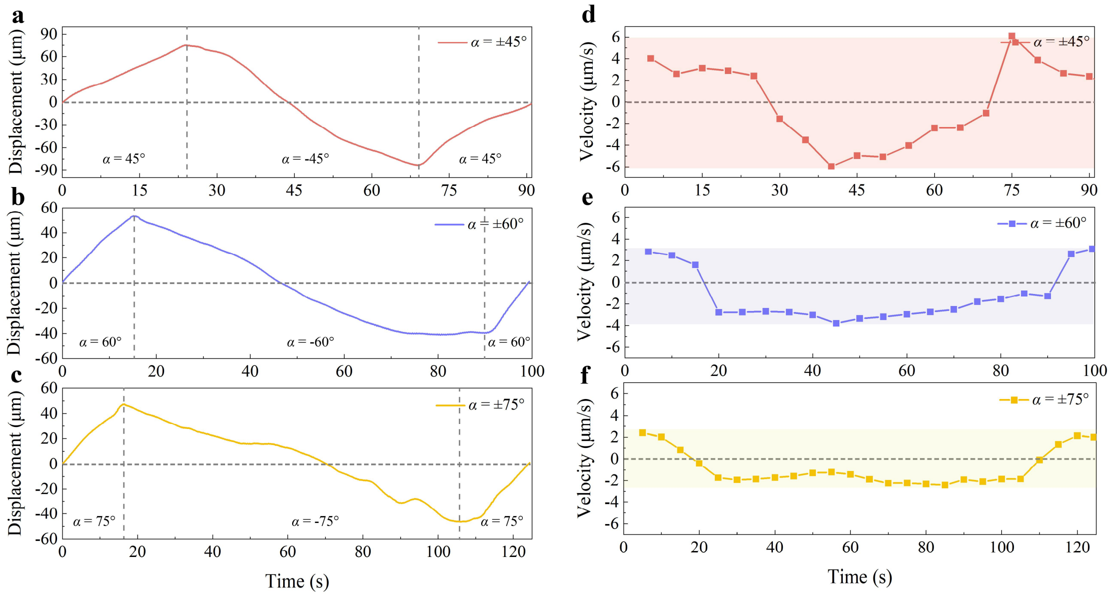

Supplementary Figure 13. (**a-c**) The variations of the lateral displacement with time when the polarization angle switches circularly between different polarization angles. (**d-f**) The variations of the lateral velocity with time when the polarization angle switches circularly between different polarization angles.

Supplementary References

1. Chaumet, P. C. & Nieto-Vesperinas, M. Time-averaged total force on a dipolar sphere in an electromagnetic field. *Opt. Lett.* **25**, 1065-1067 (2000).
2. Novotny, L. & Hecht, B. *Principles of Nano-Optics* (Cambridge University, 2006).
3. Sukhov, S., Kajorndejnukul, V., Naraghi, R. R. & Dogariu, A. Dynamic consequences of optical spin–orbit interaction. *Nat. Photonics*, **9**, 809-812 (2015).
4. Abramowitz, M. & Stegun, I. A. *Handbook of mathematical functions with formulas, graphs, and mathematical tables* (US Government printing office, 1948).
5. Stamnes, J. J. *Waves in Focal Regions: Propagation, Propagation, Diffraction and Focusing of Light, Sound and Water Waves* (Bristol, England: Adam Hilger, 1986) Ch.8.
6. Erdelyi, A. *Asymptotic expansions* (Dover, New York, 1956).
7. David, R., Dobson, S. M., Tavassoli, Z., Cabezas, M. G. & Neumann, A. W. (2009). Investigation of the Neumann triangle for dodecane liquid lenses on water. *Colloids and Surfaces A: Physicochemical and Engineering Aspects*, **333**, 12-18 (2009).
8. Aveyard, R. & Clint, J. H. Liquid lenses at fluid/fluid interfaces, *J. Chem. Soc., Faraday Trans*, **93**, 1397-1403 (1997).
9. Kajorndejnukul, V., Ding, W., Sukhov, S., Qiu, C.-W. & Dogariu, A. Linear momentum increase and negative optical forces at dielectric interface. *Nat. Photonics*, **7**, 787-790 (2013).
10. Shi, Y. et al. Chirality-assisted lateral momentum transfer for bidirectional enantioselective separation. *Light: Sci. Appl.* **9**, 62 (2020).
11. Kravets, N., Aleksanyan, A. & Brasselet, E. Chiral optical Stern-Gerlach Newtonian experiment. *Phys. Rev. Lett.* **122**, 024301 (2019).
12. Svak, V. et al. Transverse spin forces and non-equilibrium particle dynamics in a circularly polarized vacuum optical trap. *Nat. Commun.* **9**, 5453 (2018).
13. Shi, Y. et al. Stable optical lateral forces from inhomogeneities of the spin angular momentum. *Sci. Adv.* **8**, eabn2291 (2022).
