# Supplementary material for: Dynamics of polarization-tuned mirror symmetry breaking in a rotationally symmetric system
Source: Nat Commun. 2024 Jul 3;15:5586. doi: 10.1038/s41467-024-49696-x (PMC11222497; doi:10.1038/s41467-024-49696-x)
Supplement: Supplementary file 3 — Description of Additional Supplementary Files [file 41467_2024_49696_MOESM3_ESM.pdf]

Description of Additional Supplementary Files

File name: Supplementary Movie 1
Description: The video shows the back-and-forth motion of a dodecane oil droplet with a radius r = 9.7μm in a linear trap when the incident light is switched between polarization angles α = -45° and α = 45°. The green arrow is the projection of the incident light on the xy-plane, along the $+x$ direction. The oil droplet moves along the $+y$ direction when polarization angle α = 45°, and moves along the $-y$ direction when α = -45°. The speed of the movie is 10 times faster.

File name: Supplementary Movie 2
Description: The video shows the lateral motion of a dodecane oil droplet with a radius r = 10.5μm in a linear trap when the incident light is switched between different polarization angles. The speed of the movie is 5 times faster.

File name: Supplementary Movie 3
Description: The video shows the movement of an aggregate composed of multiple oil droplets in a linear trap when the incident light is switched between polarization angles α = -45° and α = 45°. Over time, multiple oil droplets are captured in the linear trap and move simultaneously. The movement direction of the oil droplets obviously depends on the polarization direction of the linearly polarized light. The speed of the movie is 10 times faster.
